# Deciphering the regulatory role of ADAM8 in the PDAC tumor microenvironment

**DOI:** 10.1007/s13402-026-01266-7

**Published:** 2026-08-03

**Authors:** Kimia Zandieh, Lena Cook, Kai Zhao, Constanze Nagl, Yutong Gao, Pietro diFazio, Detlef K. Bartsch, Uta-Maria Bauer, Marion Meixner, Daniela Yildiz, Corinna Keber, Christopher Nimsky, Jörg W. Bartsch

**Affiliations:** 1 Department of Neurosurgery, Philipps University Marburg, Baldingerstrasse, 35033 Marburg, Germany; 2 Visceral Surgery, Philipps University Marburg, Baldingerstrasse, 35033 Marburg, Germany; 3 Institute for Molecular Biology and Tumor Research, Philipps University Marburg, Hans-Meerwein-Strasse 2, 35043 Marburg, Germany; 4 Institute for Pharmacology, Homburg University, Homburg, Germany; 5 Institute of Pathology, Philipps University Marburg, Baldingerstrasse, 35033 Marburg, Germany

**Keywords:** PDAC, Tumor microenvironment, ADAM8, STAT3, FAK, Src, Macrophage, Neutrophil

## Abstract

**Background:**

Pancreatic ductal adenocarcinoma (PDAC) is an aggressive malignancy with limited therapeutic options, driven in part by its immunosuppressive tumor microenvironment (TME). Tumor-associated macrophages (TAMs) and neutrophils (TANs) contribute to tumor progression and immune evasion. A Disintegrin and Metalloproteinase 8 (ADAM8), a zinc-dependent protease, is strongly upregulated in PDAC and correlates with poor clinical outcomes, suggesting a regulatory role in tumor progression.

**Methods:**

Wild-type (WT) and Adam8 knockout (A8KO) PDAC cell lines were generated using the CRISPR-Cas9 technique, and PDAC mouse models with or without Adam8 expression were established to investigate the role of ADAM8 in tumor and immune cells. In vitro assays, including Western blotting, qPCR, migration and invasion assays, proliferation assays, ELISA, cytokine and proteome analyses, as well as co-culture experiments with PDAC cells and either macrophages or neutrophils, were employed to assess the effects of ADAM8 on tumor–immune cell crosstalk. In parallel, in vivo WT and A8KO KPC models were generated, genotyped, and monitored to evaluate the impact of ADAM8 on survival, tumor growth, and immune cell recruitment within the PDAC TME.

**Results:**

ADAM8 deletion reduced tumor cell proliferation and migration, associated with reduced activation of FAK/Src/STAT3 signaling and altered secretion of cytokines including GM-CSF, M-CSF, ICAM-1, and TNF-α. Co-culture assays demonstrated that ADAM8 enhanced reciprocal signaling between tumor cells and TAMs/TANs, promoting pro-oncogenic activation. Migration assays and in vivo analyses revealed that ADAM8 facilitated recruitment of macrophages and neutrophils in PDAC TME, while *Adam8*KO tumors exhibited reduced immune infiltration and altered macrophage polarization.

**Conclusion:**

Our findings demonstrate that ADAM8 promotes PDAC aggressiveness by enhancing tumor cell proliferation and migration, activating FAK/Src/STAT3 signaling, and driving macrophage and neutrophil recruitment through cytokine regulation. By orchestrating both tumor-intrinsic pathways and tumor–immune interactions, ADAM8 emerges as a key determinant of PDAC progression and a systemic target for therapeutic intervention.

**Supplementary Information:**

The online version contains supplementary material available at 10.1007/s13402-026-01266-7.

## Introduction

Pancreatic ductal adenocarcinoma (PDAC) is classified among the most life-threatening malignancies globally, with only about 11% of patients surviving five years after diagnosis. Due to its rising incidence, it is expected to be the second major cause of cancer mortality in the United States by the year 2030 [1]. A defining feature of PDAC is its fibrotic and complex tumor microenvironment (TME), which comprises extracellular matrix (ECM) components, myeloid cells, peripheral nerves, and cancer-associated fibroblasts (CAFs). This desmoplastic stroma contributes not only to physical barriers against drug delivery but also to an immunosuppressive tumor niche [2, 3].

Immune cells exhibit a dual role in PDAC development. Early infiltration of cytotoxic T cells and natural killer (NK) cells may have anti-tumor effects; however, in advanced PDAC the dominant immune cell landscape is largely shifted to an immunosuppressive profile. Tumor-associated macrophages/neutrophils, myeloid-derived suppressor cells and regulatory T cells interact with tumor cells and exchange cytokines and matrix remodelling factors, which influence immune cell behavior [4–6]. These tumor-promoting immune cells secrete cytokines such as IL-6, GM-CSF, TGF-β and M-CSF, which promote cancer cell proliferation, neovascularization, and immune evasion [6–8]. Notably, in vivo research demonstrated that GM-CSF released by tumors can act as a chemoattractant for myeloid cells, and eliminating this cytokine can create an immunosuppressive niche, facilitating PDAC progression [9].

Among the molecules involved in PDAC development and immune modulation, A Disintegrin and Metalloprotease 8 (ADAM8) is an essential player. ADAM8 is a member of the ADAM family of membrane-bound metalloproteases, and consists of several functional domains: a prodomain, a metalloproteinase domain, a disintegrin-like region, a cysteine-rich segment, an epidermal growth factor (EGF)-like domain, a transmembrane region, and a cytoplasmic tail. Clinical evidence indicates that elevated levels of ADAM8 expression adversely impact patient outcomes, including shorter survival times and a higher likelihood of tumor invasion [10, 11].

Although ADAM8 has been more extensively studied for its activity as an extracellular sheddase, recent evidence highlights the significance of its intracellular (cytoplasmic) domain. This domain can interact with intracellular proteins and activate multiple signaling pathways, including focal adhesion kinase (FAK), Signal Transducer and Activator of Transcription 3 (STAT3) and Extracellular signal-regulated kinases (ERKs) [12, 13]. In advanced PDAC, FAK activity is often elevated and contributes to the restructuring of the tumor stroma, which results in enhanced tumor growth and resistance to chemotherapy [14]. Studies revealed that dysregulated activation of STAT3 correlates with increased tumor progression, neovascularization, and invasiveness. For instance, atypical STAT3 signaling can modulate VEGF and MMP-9 levels, leading to higher angiogenesis and cancer dissemination [15].

Beyond ADAM8 tumor-intrinsic roles, a study of immune cell populations in tumor tissues of PDAC patients reported ADAM8 expression not only in cancer cells but also in neutrophils, macrophages and natural killer (NK) cells [16]. Furthermore, ADAM8 can regulate the secretion of cytokines and chemokines involved in immune cell migration. As an example, in a glioblastoma model, ADAM8 activity was determined to upregulate CCL2 production, and thereby enhance macrophage infiltration into the TME [17].

While prior studies have demonstrated ADAM8 involvement in promoting tumor cell invasiveness, there remains a considerable gap in understanding how ADAM8 expression in both tumor and immune cells contributes to the communication of PDAC cells and infiltrating immune cells via intracellular signaling cascades. Therefore, we aimed to investigate the impact of ADAM8 deficiency on tumor–immune interactions with the focus on exchanging cytokines, immune cell recruitment, and downstream activation of FAK/Src/STAT3 pathways.

## Materials and methods

### Cell culture

KPC3 mouse PDAC cell line and primary bone marrow isolated cells were cultured in DMEM (Gibco^TM^, Life Technologies, Carlsbad, CA, USA) supplemented with 10% (v/v) Fetal Bovine Serum (FBS) (Sigma-Aldrich, Munich, Germany), 0.1 mg/mL Penicillin-streptomycin (GibcoTM, Life Technologies), 1 mM Sodium Pyruvate (Gibco^TM^, Life Technologies, Carlsbad, CA, USA) and 0.1 mg/mL Penicillin-Streptomycin (GibcoTM, Life Technologies, Carlsbad, CA, USA) in a humidified incubator with 5% CO2.

### Generation of *Adam8* knockout KPC3 cells using CRISPR/Cas9 technique

To establish *Adam8*-deficient KPC3 cells, the ADAM8 Mouse Gene Knockout Kit (OriGene, Rockville, ML, USA) was used. Briefly, 500,000 KPC cells were seeded, and two transfection preparations with different guide RNAs (gRNAs) were prepared. For this purpose, 2.5 μg of each of the expression vectors was pipetted together with 125 μl OPTIMEM and 2.5 μl Plus Reagent. In two further 1.5 mL reaction tubes, 125 μl OPTIMEM and 7.5 μl Lipofectamine LTX Reagent were added. After combining the two preparations (expression vector preparation and Lipofectamine preparation), a 5-minute incubation at RT was done. The combined preparations were then dripped onto the cells and incubated at 37 °C. On the third day, the cells were transferred to a 10 cm dish and further incubated at 37 °C. Then, transfected KPC cells were passaged according to the manufacturer’s instructions. Single-cell clones were generated subsequent to puromycin selection (1 μg/mL) (InvivoGen, San Diego, CA, USA).

### Animal experimentation

All animal experiments (breeding, genotyping, cell isolations, tumor monitoring, and phenotyping) described here are according to the Tierschutzgesetz and authorized by local authorities (Regierungspräsidium Gießen) under file name G_86/2018 to JWB.

### Generation of global *Adam8* knockout mice

*Adam8* knockout mice were generated according to what has been earlier described [18]. *ADAM8*^−/−^ mice with the mixed genetic background of 129/Ola; C57/BL/6 were continuously crossed with C57/BL/6 mice for more than 10 generations to minimize genetic variance in *A8KO* C57/BL/6 mice.

### Harvesting of bone marrow cells from mice

Briefly, bone marrow cells were isolated from WT/*A8*KO mice by dissecting femurs and tibias post-euthanasia under approved ethical protocols. Bones were washed in ice-cold PBS, and marrow flushed with supplemented DMEM through a 100 µm cell strainer. The cell suspension was centrifuged at 400 × g for 7 minutes at 4 °C. Red blood cells were lysed using a Red Blood lysis solution (Miltenyi Biotech, Bergisch Gladbach, Germany), followed by another centrifugation and a final wash with supplemented DMEM before downstream use.

### Preparation of bone marrow-derived macrophages

The harvested bone marrow cells from WT and *A8KO* C57BL/6 mice were seeded in normal growth medium (mentioned previously) supplemented with 20 ng/ml recombinant M-CSF protein (Peprotech, Cranbury, NJ, USA) for 48 hours in a humidified incubator with 5% CO2. The cell culture medium was refreshed for an additional 48 hours.

### Neutrophil isolation from mouse bone marrow

The extracted bone marrow cells from WT and A8KO C57BL/6 mice were dissolved in normal growth medium (mentioned previously) supplemented with 10% FBS. Neutrophil isolation was performed using the Neutrophil isolation kit and through magnetic separation with LS columns (Miltenyi Biotech, Bergisch Gladbach, Germany) according to the manufacturer’s protocol. Cell suspensions were centrifuged at 300 × g for 10 minutes, and pellets were resuspended in buffer. A biotin-conjugated neutrophil antibody cocktail and Anti-Biotin MicroBeads were sequentially added, with incubations at 2–8°C, followed by washing steps. The labeled cells were then applied to pre-equilibrated LS Columns placed in a magnetic separator (Miltenyi Biotech, Bergisch Gladbach, Germany). Unlabeled neutrophils were collected from the flow-through and wash fractions, representing the enriched neutrophil population. The cells were cultured in DMEM (Gibco^TM^, Life Technologies, Carlsbad, CA, USA) supplemented with 10% (v/v) Fetal Bovine Serum (FBS) (Sigma-Aldrich, Munich, Germany), 0.1 mg/mL Penicillin-streptomycin (GibcoTM, Life Technologies), 1 mM Sodium Pyruvate (Gibco^TM^, Life Technologies, Carlsbad, CA, USA) and 0.1 mg/mL Penicillin-Streptomycin (GibcoTM, Life Technologies, Carlsbad, CA, USA) in a humidified incubator with 5% CO2.

### Co-culture of KPC3 cells with bone marrow-derived macrophages

In the upper chamber of a ThinCert™ Cell Culture Insert (0.4 µm pore size), 500,000 WT or A8KO KPC3 cells were placed, while an equal number of bone marrow–derived macrophages were added to the lower surface. The co-cultivation was maintained for 48 hours, after which cells were collected for RNA and protein extraction, followed by qPCR and Western blot analysis.

### Co-culture of KPC3 cells with bone marrow isolated neutrophils

600,000 WT/A8KO KPC3 cells were seeded in the upper part of a ThinCertTM Cell Culture Insert with a 0.4 µm diameter (Greiner Bio-One GmbH, Frickenhausen, Germany). 600,000 bone marrow isolated neutrophils were seeded in the lower compartment (well) and co-cultured for 16 hours. Cells were either harvested for RNA isolation or protein extraction and subsequent qPCR and Western blot analysis.

### Scratch assay on PDAC cells

In a 24-well plate, 70,000 cells were introduced into each compartment of a Culture-Insert 2 Well (ibidi GmbH, Gräfelfing, Germany) and left to attach for around 6 hours. Next, the standard growth medium was replaced with DMEM supplemented with 0.5% (v/v) FBS to induce overnight starvation. The next day, the inserts were taken out, and the starvation medium was replaced by a fresh normal growth medium for untreated WT/*A8KO* KPC3 cells, and with the conditioned media from WT/*A8KO* macrophages for other wells. Then, images of scratch edges between the cells at time points of 0 and 24 hours were taken and analysed by ImageJ software

### Migration assay on macrophages

70,000 macrophages per well of a Culture-Insert 2-well were seeded in a 6-well plate with normal growth medium and cultured for 1 day. The following day, the 2-well inserts were removed, and a ThinCertTM Cell Culture Insert with a 0.4 µm diameter was placed on the wells to treat the cells with different conditions in the upper compartment, including 70,000 WT/*A8KO* KPC3 cells, 50 ng/ml murine recombinant GM-CSF, M-CSF, TNF-α (Peprotech, Hamburg, Germany) and ICAM-1 (Biolegend, San Diego, CA, USA) proteins in normal medium. Afterwards, pictures of the wound margins at time points of 0 and 24 hours were captured and analysed using ImageJ software.

### Neutrophil migration assay

50,000 neutrophils were seeded on the ThinCertTM Cell Culture Inserts with an 8 µm pore size (Greiner Bio-One GmbH, Frickenhausen, Germany) in DMEM medium supplemented with 0.5% (v/v) FBS. On the lower part of the insert, different conditions were applied, including WT/*A8KO* KPC3 cells-derived supernatant, 50 ng/ml murine recombinant GM-CSF, TNF-α (Peprotech, Hamburg, Germany), ICAM-1, TREM-1 (R&D Systems, Minneapolis, MN, USA) proteins; 100 ng/ml GM-CSF and TNF-α neutralizing antibodies (R&D Systems, Minneapolis, MN, USA) in DMEM medium supplemented with 10% (v/v) FBS. Neutrophils were incubated for 16 hours at 37 °C with 5% CO2. Subsequently, the migrated cells were collected from the lower part and counted.

### Cell viability assay

Either KPC3 cells or macrophages (5,000 of each per well) were plated in triplicate in a 96-well plate. For KPC3 cells, viability was assessed at 9, 24, 36, 48, and 56 hours, while for macrophages, measurements were taken between days 5 and 8. In each case, 50 µl of CellTiter-Glo® 3D Cell Viability Assay solution (Promega, Walldorf, Germany) was mixed, the samples were shaken for 15 minutes and subsequently incubated in darkness at room temperature for 15 minutes. Luminescence was then recorded using a multiwell plate reader (FLUOstar OPTIMA, BMG Labtech, Offenburg, Germany).

### Inhibiting apoptosis and autophagy assay

To assess the autophagy and apoptosis rates of PDAC cells, 5000 KPC3 cells were placed in triplicate in a multi-well plate and incubated for 1 hour to attach. The procedure was followed by treating the cells with 100 nM, 500 nM and 1 µM apoptosis inhibitor (Q-VD-OPH) (Medchemexpress, NJ, USA); and 10 µM and 100 µM of autophagy inhibitor (3-Methyladenine (3-MA) (Houston, TX, USA)) for 24 h and 72 h. Subsequently, 50 µl of CellTiter-Glo® 3D Cell Viability Assay solution was added to the cells, and the procedure continued as described above (Cell viability assay section).

### Invasion assay

For evaluating the invasiveness of KPC3 cells, ThinCertTM Cell Culture Inserts with an 8 µm pore diameter were used. Firstly, A 50 µl layer of Growth Factor-Reduced Matrigel Basement Membrane Matrix (Corning®, Corning, NY, USA) was applied to the upper surface of the Thincert. and incubated for 1 hour to harden. Afterwards, 50,000 cells were seeded in 50 µl DMEM Medium supplemented with 0.5% (v/v) FBS on the lower surface of the Thincert. Cells were incubated for 4 hours to attach to the membrane. Therafter, 250 µl of DMEM supplemented with 20% (v/v) FBS was added to the upper chamber of the Thincert, while 750 µl of DMEM containing 0.5% (v/v) FBS was placed in the lower well. The setup was incubated for 24 hours at 37 °C. The next day, cells were fixed with 4% (w/v) PFA for 30 minutes and subsequently permeabilized with 0.3% (v/v) Triton X for an additional 30 minutes. Nuclear staining was performed overnight using Hoechst 33,342 dye (Thermo Scientific, Waltham, MA, USA). Z-stack images were acquired from several randomly selected fields, and cell populations were quantified with ImageJ software by distinguishing non-invasive cells located on the underside of the Thincert from invasive cells that had migrated into the Matrigel layer.

### Mouse proteome array

Briefly, the secretion level of 40 different cytokines and chemokines in the cell culture supernatant of WT/*A8*KO KPC3 cells, bone marrow-derived macrophages and neutrophils was evaluated using the Mice Proteome Array (ARY006, R&D Systems, Minneapolis, MN, USA; see Figure S1 for annotation of analytes). The array was carried out according to the manufacturer’s protocol, and subsequently, the membranes were detected using the ChemiDoc MP Imaging System (Bio-Rad Laboratories GmbH). The mean pixel density of the resulting dots was evaluated using ImageJ software.

### Protein extraction and western blot analysis

Cells were harvested for protein extraction, resuspended in RIPA buffer (50 mM HEPES pH 7.4; 150 mM NaCl; 1% (v/v) NP-40; 0.5% (w/v) Natriumdeoxycholate; 0.1% (w/v) SDS; 10 mM Phenantrolin; 10 mM EDTA; PierceTM Protease Inhibitor and PierceTM Phosphatase Inhibitor (Thermo Scientific, Waltham, MA, USA)) and incubated for 30 min on ice. This process was followed by a centrifugation at 12,000 g for 5 min at 4 °C. The protein concentration of the cell lysates was quantified by Pierce TM BCA Protein Assay Kit (Thermo Scientific, Waltham, MA, USA). Proteins from equivalent quantities of lysate were heated at 95 °C for 5 minutes and separated according to their molecular weight by using sodium dodecyl sulfate–polyacrylamide gel electrophoresis (SDS-PAGE). The proteins were subsequently transferred onto a nitrocellulose membrane (GE Healthcare, Chicago, IL, USA) and blocked with a solution of 5% non-fat dry milk in 1× TBST (50 mM Tris, pH 7.5; 150 mM NaCl; 0.1% (w/v) Tween-20) for 1 hour on a shaker. The membrane was then incubated overnight at 4 °C with the primary antibodies including: anti-ADAM8 (1:400; kindly provided by Dr. Daniela Yildiz, Homburg University, Germany), anti-β-TUBULIN (1:2000; NB600-936, Novus Biological, Littleton, CO, USA), anti-GAPDH (1:10,000; ab181602, Abcam, Cambridge, UK), anti-P-STAT3 (Tyr705) (1:2000; 9145, Cell Signaling Technology, Danvers, MA, USA), anti-STAT3 (1:1000; 8768, Cell Signaling Technology, Danvers, MA, USA), anti-Src (1:1000; 36D10, Cell Signaling Technology, Danvers, MA, USA), anti-P-Src (Tyr416) (1:1000; CD49G4, Cell Signaling Technology, Danvers, MA, USA), anti-FAK (1:1000; 3285, Cell Signaling Technology, Danvers, MA, USA) and anti-P-FAK (Tyr397) (1:1000; 3283, Cell Signaling Technology, Danvers, MA, USA). Afterwards, the membranes were washed 3 times with TBST and exposed to HRP-conjugated secondary antibodies (1:5000; Abcam, Cambridge, UK) for 1 h, followed by another round of washing steps. Lastly, proteins were detected using Chemiluminescent HRP Substrate, Western Bright Sirius (Advansta Inc., San Jose, CA, USA) and the ChemiDoc MP Imaging System (Bio-Rad Laboratories GmbH).

### RNA isolation and quantitative real-time PCR

To extract total RNA, 1 ml of QIAzol reagent (Qiagen, Hilden, Germany) was added to the seeded cells, followed by chloroform-induced phase separation and incubation at room temperature for 3 min and subsequent centrifugation at 12,000 g for 15 min at 4 °C. Next, 500 μl of Isopropanol was added to the collected upper phase to obtain the RNA pellet. After centrifugation, the pellet was washed with 75% ethanol, air-dried, and resuspended in DEPC-treated water. RNA yield and purity were assessed with a.NanoPhotometer® NP80 (Implen, Munich, Germany). For cDNA synthesis, purified RNA was processed with the EcoDry™ RNA to cDNA kit (Takara Bio Inc., Kusatsu, Japan) to conduct reverse transcription according to the manufacturer’s instructions. Quantitative real-time PCR was performed using iTaqTM Universal SYBR® Green Supermix (Bio-Rad Laboratories GmbH, Feldkirchen, Germany). For quantitative real-time PCR, reactions (20 µl total volume) were prepared using iTaq™ Universal SYBR® Green Supermix (Bio-Rad Laboratories GmbH, Feldkirchen, Germany). Each reaction contained cDNA equivalent to 20 ng of RNA and the appropriate primers. Amplification was performed on a StepOnePlus™ Real-Time PCR System (Thermo Fisher Scientific, Waltham, MA, USA). The ΔCt method was applied to evaluate relative mRNA expression with XS13 used as the reference gene in all cell types except neutrophils, where GAPDH served as a control. The primers were as follows: Adam8 QuantiTect Primer Assay (Qiagen, Hilden, Germany), XS13 (ribosomal RPLP0 gene) (fw: 5’-TGG GCA AGA ACA CCA TGA TG-3’; rev: 5’-AGT TTC TCC AGA GCT GGG TTG T-3’), Icam-1 (fw: 5’- CAA TTT CTC ATG CCG CAC AG-3′; rev: 5′-AGC TGG AAG ATC GAA AGT CCG-3′), Tnf-α (fw: 5’-CCA CAT CTC CCT CCA GAA AAG A-3’; rev: 5’- GCT GGG TAG AGA ATG GAT GAA C-3’), M-csf (fw: 5′-AGT ATT GCC AAG GAG GTG TCA G-3′; rev: 5′-ATC TGG CAT GAA GTC TCC ATT T-3′), Stat3 (Fw: 5’-TGT CTC CAC TTG TCT ACC T-3’; rev: 5’-CAG CAC CTT CAC CGT TAT-3’), Gapdh (Fw: 5’- CAT CAC TGC CAC CCA GAA GAC TG-3’; rev: 5’-ATG CCA GTG AGC TTC CCG TTC AG-3’).

### Enzyme-linked immunosorbent assay (ELISA)

Soluble levels of Gm-CSF in KPC3 cells, neutrophil and macrophage-derived supernatants were determined by DuoSet ELISA kit (DY415-05, R&D Systems, Minneapolis, MN, USA). The supernatants from the co-culture experiments of WT/*A8*KO KPC3 cells with either WT/A8KO macrophages or WT/*A8*KO neutrophils were collected and centrifuged at 400 g for 7 min and assessed by the ELISA kit according to the manufacturer’s instructions.

### A8KO KPC mouse generation and scoring

To investigate ADAM8-dependent interactions of tumor cells with the microenvironment in vivo, well-established mouse models of pancreatic cancer (*Kras*^*LSL-G12D*^, *Trp53*^*R172H/+*^, *Pdx*^*Cre/+*^(KPC) mice) were utilized and crossed to global *Adam8* knockout on the mouse background strain C57Bl/6J. A8 WT/KO mice were examined weekly to monitor tumor development. Palpable masses of the pancreas were investigated by ultrasound (Vevo 2100, VisualSonics) to confirm the presence of tumors and to determine tumor volumes. The mice that reached the endpoint criteria were sacrificed and utilized for survival analysis and histological experiments.

### Immunohistochemistry assay

To confirm the lack of ADAM8 expression in A8KO pancreas tissues from A8KO mice (with WT mice as controls), and to compare the macrophage and neutrophil infiltration as well as M1 and M2 polarization state of macrophages between WT and A8KO mice pancreatic tissues, immunohistochemical analyses on formalin-fixed, paraffin-embedded (FFPE) pancreatic tissues were performed. Tissue sections from WT and A8KO mice were stained using anti-ADAM8 (1:400; kindly provided by Dr. Daniela Yildiz, Homburg University) and a standard VectaStain protocol. Routine pathology was performed by PAS staining, IHC for pan-cytokeratin (CKMNF116, Hoelzel Diagnostica, Köln, Germany) and phospho-STAT3 (anti-P-STAT3 (Tyr705), 1:200; 9145, Cell Signaling Technology, Danvers, MA, USA). To assess the macrophage identification and M1/M2 phenotyping, double staining on the mentioned tissues using Rat anti-mouse F4/80 antibody (1:1000; MCA497G; Bio-Rad Laboratories GmbH, Feldkirchen, Germany) as a mouse macrophage marker, anti-iNOS antibody (1:500; ab3523, Abcam, Cambridge, UK) as an M1 marker and anti-liver Arginase antibody (1:1000; ab91279, Abcam, Cambridge, UK) as an M2 marker was performed. Additionally, to detect and compare neutrophil levels in the mice tissues, anti-Human/Mouse Myeloperoxidase/MPO antibody (1:100; AF3667, R&D Systems, Minneapolis, MN, USA) was utilized. The protocol for staining of macrophages and neutrophils is as follows. Briefly, slides were deparaffinized by heating to 60 °C for 1 h and xylene treatment and afterwards rehydrated through graded ethanol washes. Antigen retrieval was carried out utilizing Target Retrieval Solution TRS Citrate pH 6.0 (S 236,984-2, Dako, Waldbronn, Germany) for 30 min. To block endogenous peroxidase activity, sections were treated with Peroxidase Blocking Solution (S202386-2, Dako, Waldbronn, Germany). The slides were exposed to primary antibodies (mentioned above) for 45 min, subsequently treated with biotinylated secondary antibodies, and afterwards with avidin-biotin complex (ABC Elite Kit) (PK-6100, Vector Labs, Burlingame, CA, USA) for 30 min. Then, the stainings were visuallized using Dako DAB peroxidase substrate kit (K500711-2, Dako, Waldbronn, Germany). To do co-staining, in addition to DAB, HRP Magenta Substrate Chromogen was used. Finally, nuclear staining was carried out with Hematoxylin, followed by dehydration using an increasing ethanol gradient and xylene washes. All staining procedures after antigen retrieval were accomplished with the Dako Cytomation system Autostainer Plus.

### DNA extraction, PCR and electrophoresis of mouse biopsies

To identify the genotype of the WT/*A8*KO as well as WT/A8KO KPC mice, DNA from ear biopsies of the mentioned mice was isolated using peqGOLD Blood & Tissue DNA Mini Kit (13–3396-02 VWR LIFE SCIENCE, Darmstadt, Germany) according to the manufacturer’s instructions. A standard PCR reaction system was prepared and performed using following primers: Adam8 (MS204: 5´-GCA AGT GGT GGC ATT GCA GCA G-3, MS209: 5´-GAT CTG GAC GAA GAG CAT CAG-3´, MS211: 5´-CTG GCC TCC CTC ACA GTA CAG-3´ and MS213: 5´-CAC TGT TGG ACT GGC TAA GGT G-3´), Kras (fw: 5´-CTA GCC ACC ATG GCT TGA GT-3´; rev: 5´-TCC GAA TTC AGT GAC TAC AGA TG-3´) Trp53 (fw: 5´- AGC TAG CCA CCA TGG CTT GAG TAA GTC TGC-3´; rev: 5´- CTT GGA GAC ATA GCC ACA CTG-3´) and Cre (fw: 5´- AAC ATG CTT CAT CGT CGG-3´; rev: 5´- TTG CCC CTG TTT CAC TAT CCA G-3´). Subsequently, PCR products were separated by electrophoresis on a 1.5% Agarose gel and visualized to determine the genotypes of the mice.

### Statistical analysis

For statistical analysis, Student’s t-test (2 groups) or ANOVA (>2 groups) was applied. Data were considered not significant (*p* > 0.05, no asterisk), significant * (*p* < 0.05), highly significant ** (*p* < 0.01) and very highly significant *** (*p* < 0.001) or **** (*p* < 0.0001), and are expressed as the mean ± S.D.

## Results

### *Adam8* deficiency impairs migration, proliferation, and cytokine secretion in PDAC cells

**Fig. 1:**
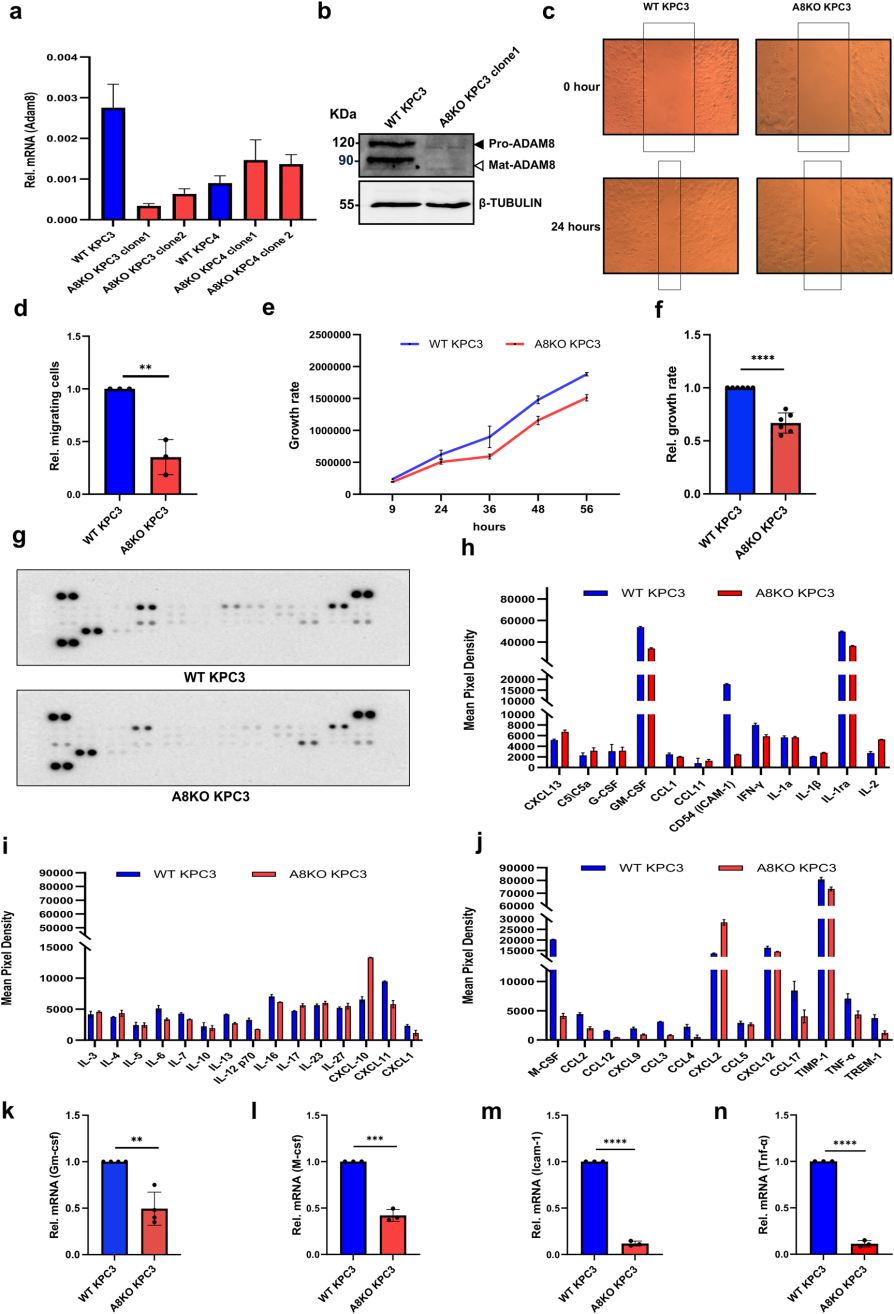
Establishment and characterization of murine WT/*A8*KO PDAC cells. (**a**) qPCR results depict mRNA expression of *A8* in WT KPC3, WT KPC4 and their corresponding CRISPR/Cas9 knockout clones. (**b**) Protein level evaluation of WT KPC3 and *A8*KO KPC3 clone 1, selected based on qPCR results, confirms the downregulation of ADAM8 (Pro-ADAM8~120 kDa, Mat-ADAM8~90 kDa) in the KO cell line; β-TUBULIN was used as a loading control (*n* = 3). (**c, d**) Scratch assay of WT/*A8*KO KPC3 cells at time points of 0 h and 24 h (*n* = 3) also (**e**) proliferation assay after 9 h, 36 h, 48 h, 56 h (*n* = 1) and maximal effect after 56 h (**f**) (*n* = 6) indicate that ADAM8 affects KPC3 cell proliferation significantly. (**g**) Dot blot analysis of proteome arrays using WT/*A8*KO KPC3-driven supernatants (h, i, j) comparing the secretion levels of 40 different cytokines and chemokines suggests that A8 influences the secretome of these cells. (**k**) Indicates relative mRNA expression of *Gm-csf* (**l**) *M-csf*, (**m**) *Icam-1* and (**n**) *Tnf-α* in WT/*A8*KO KPC3 cells (*n* = 3). Data are presented as mean values ± S.D and Student’s *t test* was used to determine significance with * *p* < 0.05, ** *p* < 0.01, *** *p* < 0.001, **** *p* < 0.0001

To investigate the role of ADAM8 in the PDAC microenvironment of mice, we initially generated some knockout clones of *Adam8* for two different KPC cell lines adapted from KPC mice (WT KPC3 and WT KPC4) using the CRISPR/Cas9 technique. To confirm the knockout (KO) of *Adam8* in these clones, we performed qRT-PCR and Western blot analysis. qPCR data displayed that endogenous *Adam8* mRNA expression is higher in WT KPC3 cells in comparison to KPC4 cells, making them more appropriate for knockout experiments. *A8* mRNA expression is downregulated in *A8*KO KPC3 clones, particularly clone 1 (Fig. 1a). Consequently, WT KPC3 and *A8*KO KPC3 clone 1 were selected for further experiments. Western blot analysis confirmed the downregulation of pro- and mature Adam8 protein in *A8*KO KPC3 clone 1 cells (Fig. 1b).

To assess the functional impact of *Adam8* knockout, a scratch assay was carried out. The quantification of the assay revealed that the deficiency of *Adam8* can reduce the migratory ability of KPC3 cells over 24 hours. (Fig. 1c, d). These results indicate that ADAM8 is important for the migratory capacity of KPC3 cells whereas proliferation under these conditions is not changed (Figure S2). Furthermore, the effect of ADAM8 on the growth rate of KPC3 cells was assessed via a proliferation assay. Growth curves displayed a consistent reduction in the proliferation rate of *A8*KO KPC3 cells compared to wild-type cells from 9 to 56 hours (Fig. 1e). Relative proliferation rate quantifications showed a statistically significant decrease in the relative growth rate of *A8*KO KPC3 cells over 24 hours (Fig. 1f). To explore whether the observed difference in growth rate resulted solely from the proliferation ability of the cells rather than autophagy or apoptosis effects, we performed an additional proliferation experiment using autophagy or apoptosis inhibitors (3-MA or Q-VD-OPH, respectively). The results demonstrated that inhibition of autophagy and apoptosis in WT/A8KO KPC3 cells over 24 and 72 hours had no notable effect on the cell numbers (Figure S3). Moreover, the invasion ability of the cells was evaluated using an invasion assay on WT/*A8*KO KPC3 cells and showed a lack of invasiveness in both cell types (Figure S4).

Besides, we conducted a proteome array on the conditioned medium from WT/*A8*KO KPC3 cells to investigate the effects of ADAM8 on cytokine secretion (Fig. 1g). Quantitative analysis showed distinct secretion patterns between the groups in some proteins; interestingly, a 40% reduction for GM-CSF, 80% for M-CSF and a 90% decrease for ICAM-1 in the KO group. Moreover, CXCL-1, IL-6, CCL2 and TNF-α were downregulated in the absence of ADAM8 (Fig.s 1h–j). Further, a qPCR analysis was performed to examine the effect of *Adam8* on mRNA expression of the interesting candidates from the array. The findings displayed lower expression of *Gm-csf* (Fig. 1k), a significant downregulation of *M-csf* (Fig. 1l), *Icam-1* (Fig. 1m) and *Tnf-α* (Fig. 1n) in *A8*KO KPC3 cells.

### Impact of ADAM8 on basal macrophage survival, migration, and secretome composition

**Fig. 2:**
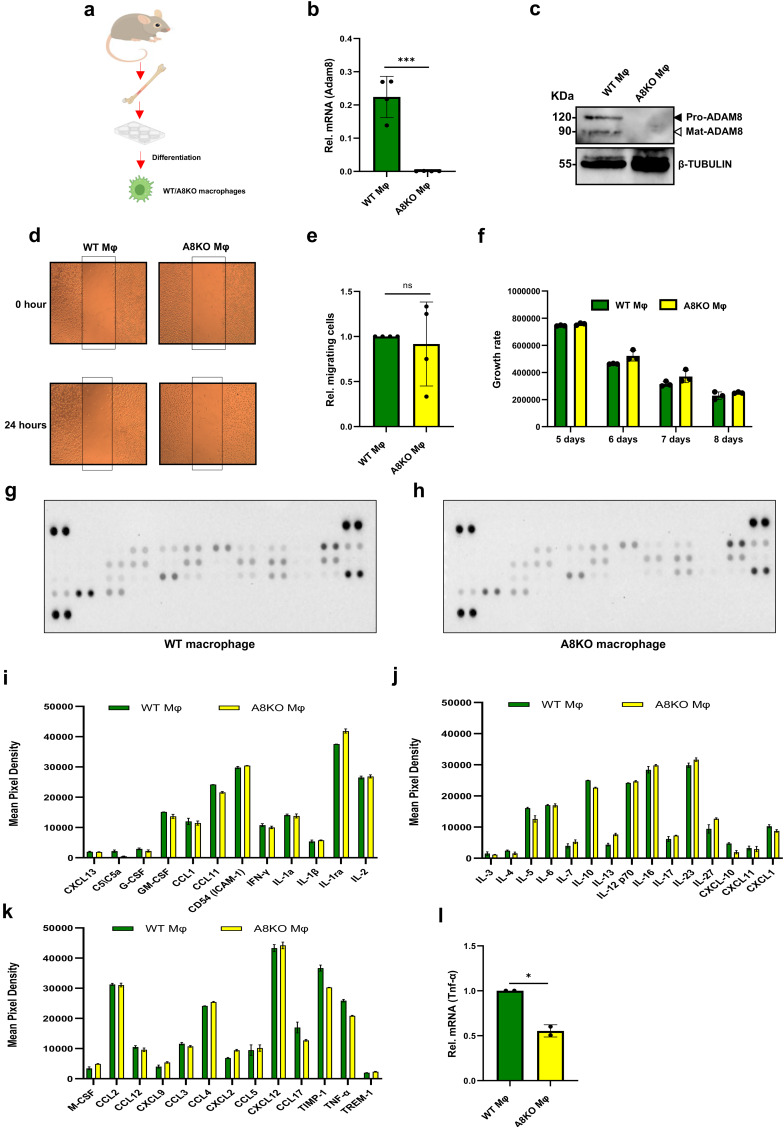
Extraction and characterization of macrophages isolated from WT/*A8*KO mice bone marrow. (**a**) The schematic model illustrates the isolation of the bone marrow cells from WT/*A8*KO mice and their differentiation into macrophages. (**b**) The qPCR analysis confirms significant downregulation of *A8* mRNA expression in *A8*KO macrophages (Mφ) (*n* = 4). (**c**) Western blot analysis further validates the absence of ADAM8 protein (Pro-ADAM8~120 kDa, Mat-ADAM8~90 ~90 kDa) in *A8*KO macrophages; β-TUBULIN served as a loading control (*n* = 3). (**d, e**) Scratch assay of WT/*A8*KO macrophages at time points of 0 h and 24 h (*n* = 4), as well as (**f**) viability assay of WT/*A8*KO macrophages at time points of 5, 6, 7 and 8 days, depict no significant difference in migration and viability between the cells. (**g, h**) Represent dot blot results from the proteome array on WT/*A8*KO-derived supernatants (i, j, k), evaluating 40 cytokines and chemokines at the secretion level. (**l**) Relative mRNA expression of *Tnf-α* in WT/*A8*KO macrophages is displayed (*n* = 2). Data are presented as mean values ± S.D, and Student’s *t test* was used with* *p* < 0.05, ** *p* < 0.01, *** *p* < 0.001

In the next step, we attempted to identify the effects of *Adam8* deficiency on bone marrow-derived macrophages. To achieve this, we extracted bone marrow cells from the WT/*A8*KO mice and differentiated them over a five-day period to macrophages (Fig. 2a). To confirm the knockout of *Adam8*, we performed a qPCR analysis that showed a significant reduction in *Adam8* mRNA expression in *A8*KO macrophages compared to WT macrophages (Fig. 2b). Western blot analysis also confirmed the absence of the pro-form and mature form of ADAM8 protein in *A8*KO macrophages (Fig. 2c).

Moreover, to evaluate the impact of ADAM8 on the migratory ability of the macrophages, we conducted a wound-healing experiment over 24 hours (Fig. 2d). The quantitative results indicated that ADAM8 has no notable effect on the migratory capacity of the macrophages (Fig. 2e). Additionally, we performed a viability assay over 5 to 8 days on WT/*A8*KO macrophages demonstrated no remarkable differences in cell viability between the two groups (Fig. 2f).

To explore the influence of ADAM8 on the secretome of the macrophages, we next performed a proteome array on the conditioned medium from WT/*A8*KO macrophages. Dot blots exhibited almost similar patterns of secretion between the groups (Fig.s 2g, h). Quantitative analysis of the cytokine secretion showed no dramatic alteration between the cells. The more interesting marker for us was TNFα, which showed a 20% reduction in KO cells (Fig. 2i–k). Finally, the mRNA expression level of *Tnf-α* was examined and demonstrated a significant downregulation in KO cells compared to the wild-type (Fig. 2l).

### Effect of ADAM8 on basal neutrophil migration and cytokine secretion

**Fig. 3:**
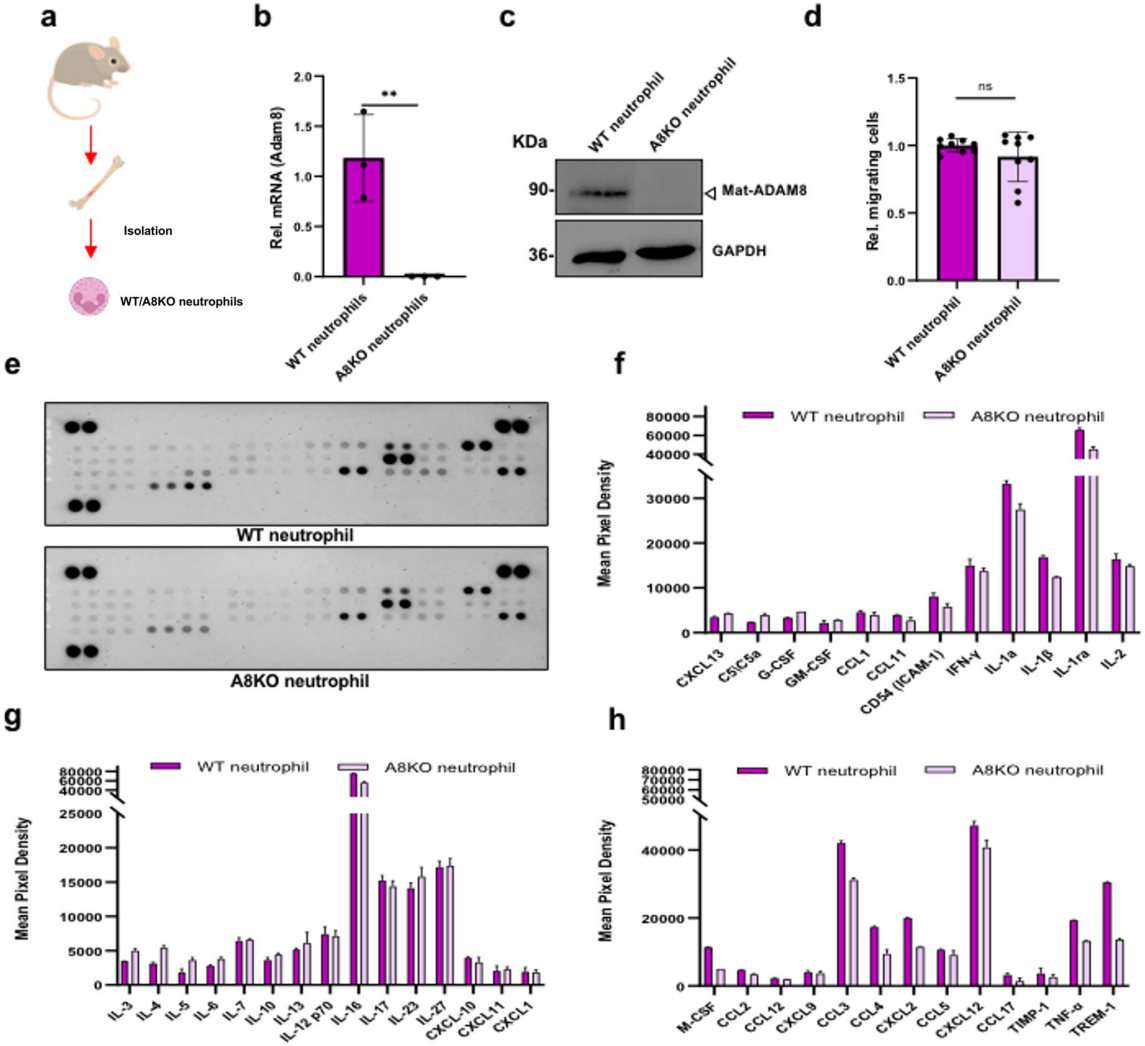
Isolation and characterization of WT/*A8*KO neutrophils from mouse bone marrow. (**a**) extraction of bone marrow from WT and *A8*KO mice and isolating neutrophils. (**b**) RNA and (**c**) protein level confirmation of A8 (Mat-ADAM8 ~90 kDa) knockout in mice; and GAPDH used as a loading control (*n* = 3). (**d**) Transwell migration assay of WT/*A8*KO neutrophils at 0 h and 16 h indicates no notable difference (*n* = 3). (**e**) Dot blot results from the proteome array on WT/*A8*KO-derived supernatants display (f, g, h) secretion levels of different cytokines and chemokines. Data are presented as mean values ± S.D, and Student’s *t test* was used with * *p* < 0.05, ** *p* < 0.01, *** *p* < 0.001

Another immune cell population we sought to characterize was WT/*A8*KO mouse-derived neutrophils. For this reason, we extracted the bone marrow from WT/*A8*KO mice and then isolated the neutrophils (Fig. 3a). To validate the *A8* knockout of the cells, we performed a qRT-PCR experiment, indicated a significant downregulation of *Adam8* in *A8*KO neutrophils compared to the wild-type ones (Fig. 3b). Protein level analysis confirmed the absence of Mat-ADAM8 protein in KO neutrophils, whereas it was detected in the wild-type ones. (Fig. 3c). Quantification of migration assay on neutrophils using transwells showed no notable difference between the WT and A8KO groups (Fig. 3d).

Next, we evaluated the cytokine production in WT/*A8*KO neutrophils using their conditioned media, respectively. Dot blots and quantifications illustrated a slight alteration between the groups in some candidates, of note, a 60% decline in M-CSF, a 40% decrease in TNF-α and a 60% reduction in TREM-1 in KO neutrophils compared to wild-type ones (Fig. 3e–h).

### Macrophages enhance Adam8-mediated FAK/Src/STAT3 signaling in PDAC cells and regulate Src/STAT3 pathways intrinsically

**Fig. 4:**
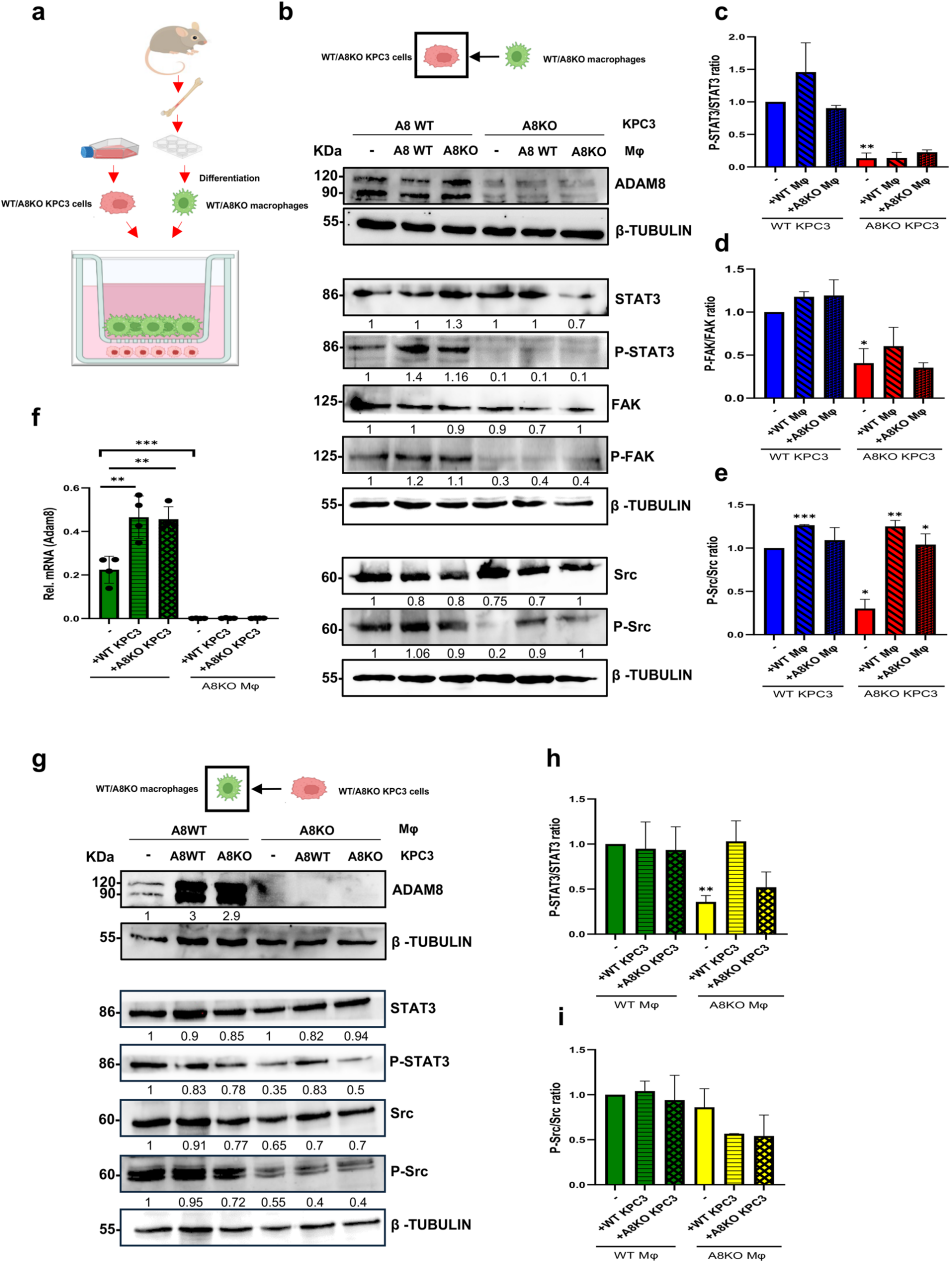
Co-culture of WT/A8KO KPC3 cells with mouse bone marrow-derived macrophages. (**a**) graphical sketch depicts the interaction between WT/*A8*KO KPC3 cells and macrophages. (**b-e**) Indicate western blot results and corresponding quantifications from the co-culture of PDAC (KPC3) cells in 6 different setups: untreated WT KPC3, WT KPC3 + WT Mφ, WT KPC3 + *A8*KO Mφ, untreated *A8*KO KPC3, *A8*KO KPC3 + WT Mφ and *A8*KO KPC3 + *A8*KO Mφ, respectively. (**f-i**) Findings from qPCR, western blot and its corresponding quantification of the co-cultivation of the macrophages under 6 different conditions: untreated WT Mφ, WT Mφ + WT KPC3, WT Mφ + *A8*KO KPC3, untreated *A8*KO Mφ, *A8*KO Mφ + WT KPC3 and *A8*KO Mφ + *A8*KO KPC3, respectively. (**b**) The protein levels of ADAM8 (*n* = 3), P-STAT3, total STAT3 as its control, (*n* = 2) P-FAK, total FAK as a control (*n* = 2), and P-Src and total Src as its control (*n* = 2) in PDAC cells before and after co-culture are presented, and β-Tubulin served as a loading control. (**c-e**) Displays the ratio of (**c**) P-STAT/STAT3, (**d**) P-FAK/FAK and (**e**) P-Src/Src protein expression in KPC3 cells after co-culture with macrophages. (**f**) shows the qPCR analysis of *Adam8* mRNA level in macrophages after co-cultivation with PDAC cells. (**g**) Western blot results of ADAM8 (*n* = 2), P-STAT3, total STAT3 as its control, P-Src and total Src as a control (*n* = 2) of macrophages before and after co-culture with KPC3 cells are displayed, and β-TUBULIN antibody is utilized as a loading control. The ratio of (**h**) P-STAT3/STAT3 and (**i**) P-Src/Src protein expression in macrophages before and subsequent to co-culture with PDAC cells are illustrated. The data in this figure are presented as mean values ± S.D, and as statistical methods, One-Way ANOVA and Student’s *t test* were used with * *p* < 0.05, ** *p* < 0.01, *** *p* < 0.001

To investigate the effects of the interaction between the tumor cells and bone marrow isolated macrophages, we conducted a co-culture experiment via a transwell assay for 48 hours (Fig. 4a). The impacts of this interaction on ADAM8 expression, its probable downstream molecules and signalling pathways were evaluated in KPC3 cells (Fig. 4b–e) and macrophages (Fig. 4f–i). STAT3, FAK and Src signaling have been observed to be critical in tumor progression, migration and invasion in some cancer entities, including pancreatic cancer [19–21]. The findings showed ADAM8 at the protein level was significantly decreased in *A8*KO KPC3 cells and was almost consistent in each group before and after co-cultivation (Fig. 4b). While total STAT3 protein showed some variations, the phosphorylated form of STAT3 (P-STAT3) was significantly downregulated in KO KPC3 cells independent of co-culture, suggesting ADAM8 has a substantial role in disrupting the STAT3 signalling pathway. Moreover, P-STAT3 in WT KPC3 cells was slightly upregulated after co-culture, demonstrating macrophage-mediated STAT3 activation. FAK protein level was mainly consistent before and after co-cultivation; however, P-FAK was notably downregulated in *A8*KO KPC3 cells and slightly upregulated in WT tumors after co-culture with macrophages. P-Src expression in the absence of ADAM8 was downregulated at translational levels from 1 to 0.2 in PDAC cells, indicating ADAM8‘s influence on impairing the Src signalling pathway. Nevertheless, macrophages could offset the observed reduction in P-Src level in KO KPC3 cells from 0.2 to 0.9 and 1 after co-culture with WT macrophages and KO macrophages, respectively (Fig. 4b). The quantitative analysis of western blot data based on the ratio of phosphorylated to total protein expression revealed a significant reduction in STAT3 (Fig. 4c), FAK (Fig. 4d) and Src activation in *A8*KO KPC3 cells. However, this effect on Src signalling was notably compensated by macrophages in KO KPC3 cells. Besides, the rate of Src activation increased after treatment of WT KPC3 cells with WT macrophages (Fig. 4e).

On the other hand, the findings indicated that macrophage-KPC3 interactions significantly enhanced A8 expression at mRNA and protein levels in macrophages (Fig. 4f, g). STAT3 at the protein level, despite some variations, remained mainly unaltered. Conversely, its activation was impaired in the lack of *Adam8* from 1 to 0.35, and this deficit was modestly compensated by signals from WT KPC3 cells. Total Src protein level showed some downregulations, particularly in KO macrophages, and its phosphorylated form was reduced to approximately half the value under all conditions in KO macrophages (Fig. 4g). Quantitative analysis of the western blot data based on the rate of phosphorylation of STAT3 and Src in macrophages depicted a significant reduction in STAT3 activation in KO macrophages and a modest upregulation following co-culture with WT PDAC cells (Fig. 4h), while Src exhibited just a slight decrease in activation in KO macrophages after co-culture (Fig. 4i).

### ADAM8 regulates endogenous Src/STAT3 signaling in PDAC cells and neutrophils, and modulates neutrophil-driven FAK activation in PDAC cells

**Fig. 5:**
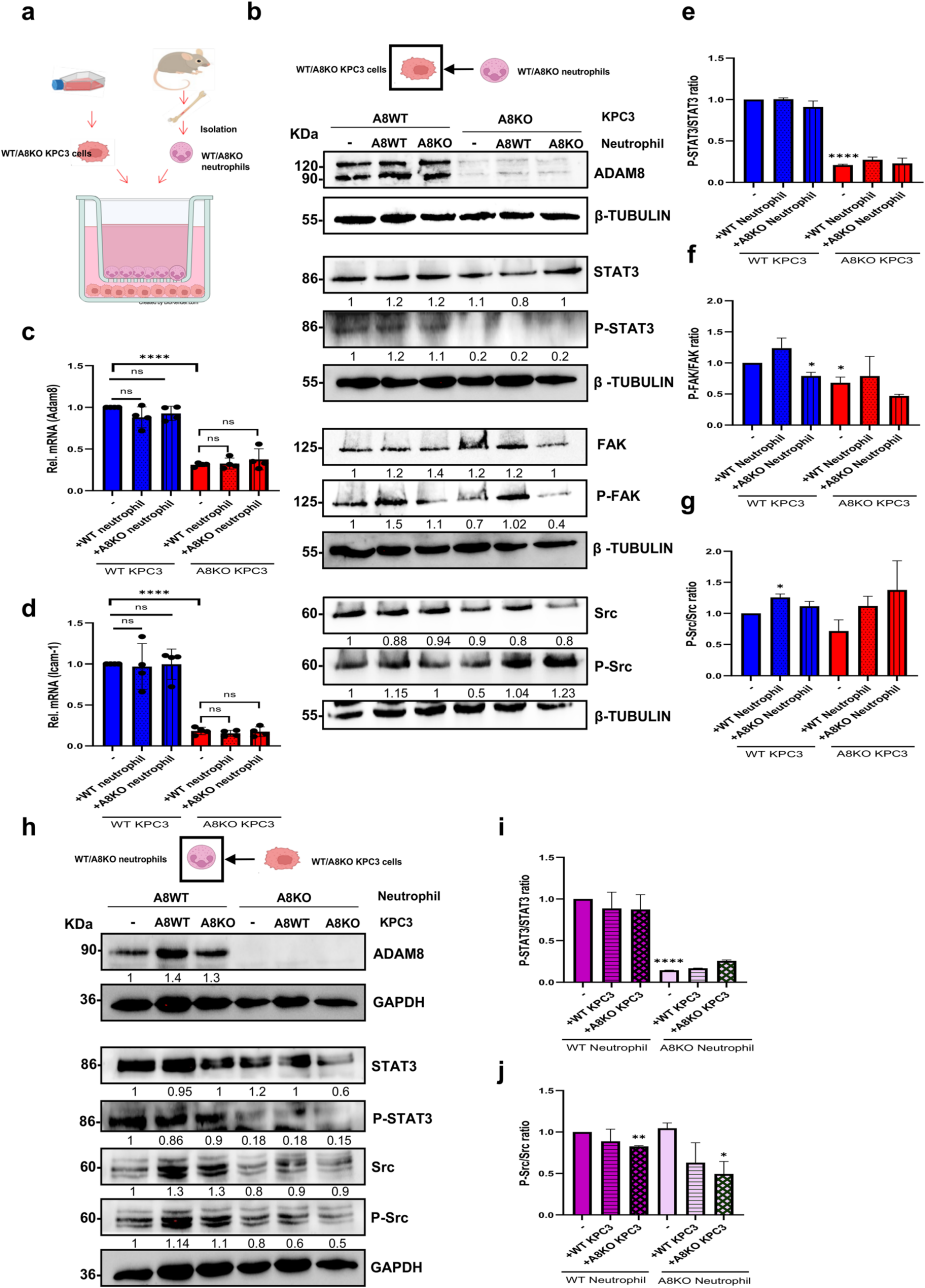
Interaction of KPC3 cells and neutrophils isolated from mouse bone marrow in a co-culture model. (**a**) The illustration shows the co-cultivation of PDAC cells and neutrophils. (**b-g**) present qPCR, western blot results and corresponding quantifications from the co-culture of the PDAC cells in 6 different setups: untreated WT KPC3, WT KPC3 + WT neutrophil, WT KPC3 + *A8*KO neutrophil, untreated *A8*KO KPC3, *A8*KO KPC3 + WT neutrophil and *A8*KO KPC3 + *A8*KO neutrophil. (**h-j**) Display western blot results and quantitative analysis from the co-culture of the neutrophils under six different conditions: untreated WT neutrophil, WT neutrophil + WT KPC3, WT neutrophil + *A8*KO KPC3, untreated *A8*KO neutrophil, *A8*KO neutrophil + WT KPC3 and *A8*KO neutrophil + *A8*KO KPC3. (**b**) Represents protein level of ADAM8 (*n* = 3), P-STAT3, total STAT3 as its control (*n* = 2), P-FAK, total FAK as its control, P-Src and total Src as a control (*n* = 3) in PDAC cells prior and subsequent to co-culture; and β-TUBULIN antibody served as a loading control. The mRNA levels of (**c**) *Adam8* (*n* = 4) and (**d**) *Icam-1* (*n* = 4) in KPC3 cells before and after co-culture. The ratio of (**e**) P-STAT3/STAT3, (**f**) P-FAK/FAK (**g**) and P-Src/Src (*n* = 2) protein expression in KPC3 cells before and after co-culture with neutrophils. The protein levels of (**h**) ADAM8 (*n* = 2), P-STAT3, STAT3 as a control (*n* = 2) and P-Src and total Src as its control (*n* = 2) in neutrophils before and after co-culture are presented, and GAPDH served as a loading control. The ratio of (**i**) P-STAT3/STAT3, (**j**) and P-Src/Src protein expression in neutrophils under the above-mentioned six conditions is shown. The data in this figure are presented as mean values ± S.D, and as statistical methods, One-Way ANOVA and Student’s *t test* were used with * *p* < 0.05, ** *p* < 0.01, *** *p* < 0.001, **** *p* < 0.0001

Considering the substantial influence of the co-culture on macrophages and tumor cells, we next examined the impacts of co-culturing neutrophils and KPC3 cells for 16 hours on the expression patterns of the same key signalling pathways on these cells using transwell assays (Fig. 5a). The results demonstrated that ADAM8 expression significantly decreased in KO KPC3 cells compared to their WT counterparts. Co-cultivation with neutrophils showed the same effect as macrophages on the PDAC cells, meaning that this interaction was not capable of modulating ADAM8 at either mRNA or protein level in PDAC cells (Fig. 5b, c). Notably, P-STAT3 level was highly reduced in KO KPC3 cells, while total STAT3, despite slight variations, remained mainly unchanged in protein (Fig. 5b). To further investigate the influence of *Adam8* depletion on intercellular signalling, FAK and Src signalling were analysed. FAK protein showed the highest value of activation in the PDAC cells co-cultivated with WT neutrophils, and the lowest phosphorylation level in *A8*KO KPC3 cells in interaction with *A8*KO neutrophils, highlighting ADAM8‘s role in activation of FAK signalling. While Total Src showed almost a similar pattern of expression in both groups, the phosphorylated form of Src (P-Src) demonstrated a lower value of activation in KO KPC3 cells, which was compensated by neutrophils from 0.5 to 1.04 under treatment with WT neutrophils and to 1.23 with KO neutrophils (Fig. 5b). The qPCR analysis on expression of *Icam*-1 as a potential target of STAT3 [13, 22] revealed a very significant downregulation of *Icam-1* in the absence of *Adam8* in PDAC cells, which was not compensated by co-culture (Fig. 5d). This pattern mirrors the behavior observed with STAT3, suggesting the possibility of *Icam-1* regulation via STAT3 signalling. Western blot analysis assessing the activation ratios of STAT3 (Fig. 5e), FAK (Fig. 5f), and Src (Fig. 5g) noticed a significant downregulation only for STAT3, but not for p-FAK and p-Src in *A8*KO PDAC cells. Notably, Src activation was to some extent restored in the presence of neutrophils.

Turning to the neutrophils, western blot analysis revealed that ADAM8 protein in WT neutrophils was slightly upregulated following co-culture with KPC3 cells (Fig. 5j). Total STAT3 protein levels, despite slight alterations in response to PDAC cells, remained largely consistent. STAT3 activation was significantly reduced in *A8*KO neutrophils from 1 to around 0.2, regardless of co-culture with tumor cells. Total and P-Src were slightly increased in WT neutrophils interacted with tumor cells; however, P-Src levels exhibited a limited decrease under KO neutrophil conditions (Fig. 5j), Finally, analysis of the rate of STAT3 and Src activation displayed a significant decrease in phosphorylation of STAT3 in KO neutrophils (Fig. 5h) and downregulation of Src activation (Fig. 5i) in WT/*A8*KO neutrophils treated with KO KPC3 cells, indicating that *Adam8* knockout impairs STAT3 and Src activation which can affect tumor-neutrophil crosstalk.

### Migration of KPC3 cells is affected by macrophages

After investigating how the interaction between KPC3 cells and macrophages can affect both cells and regulate the important tumorigenic signaling pathways in these cells, we conducted a scratch assay on WT/*A8KO* KPC3 cells to evaluate the impact of macrophage-derived factors on the motility and migratory ability of PDAC cells. We treated the tumor cells for 24 hours with conditioned media from WT/*A8KO* macrophages. The results demonstrated that the secretome of macrophages can enhance the migratory capacity of tumor cells. Notably, the findings showed that this effect was slightly more pronounced in the presence of WT KPC3 compared to the other group (Figure S5a, b). These data support the notion that macrophages in the tumor microenvironment can be tumor-promoting.

### The role of ADAM8 in PDAC cell-mediated recruitment of macrophages and neutrophils within the tumor microenvironment

**Fig. 6:**
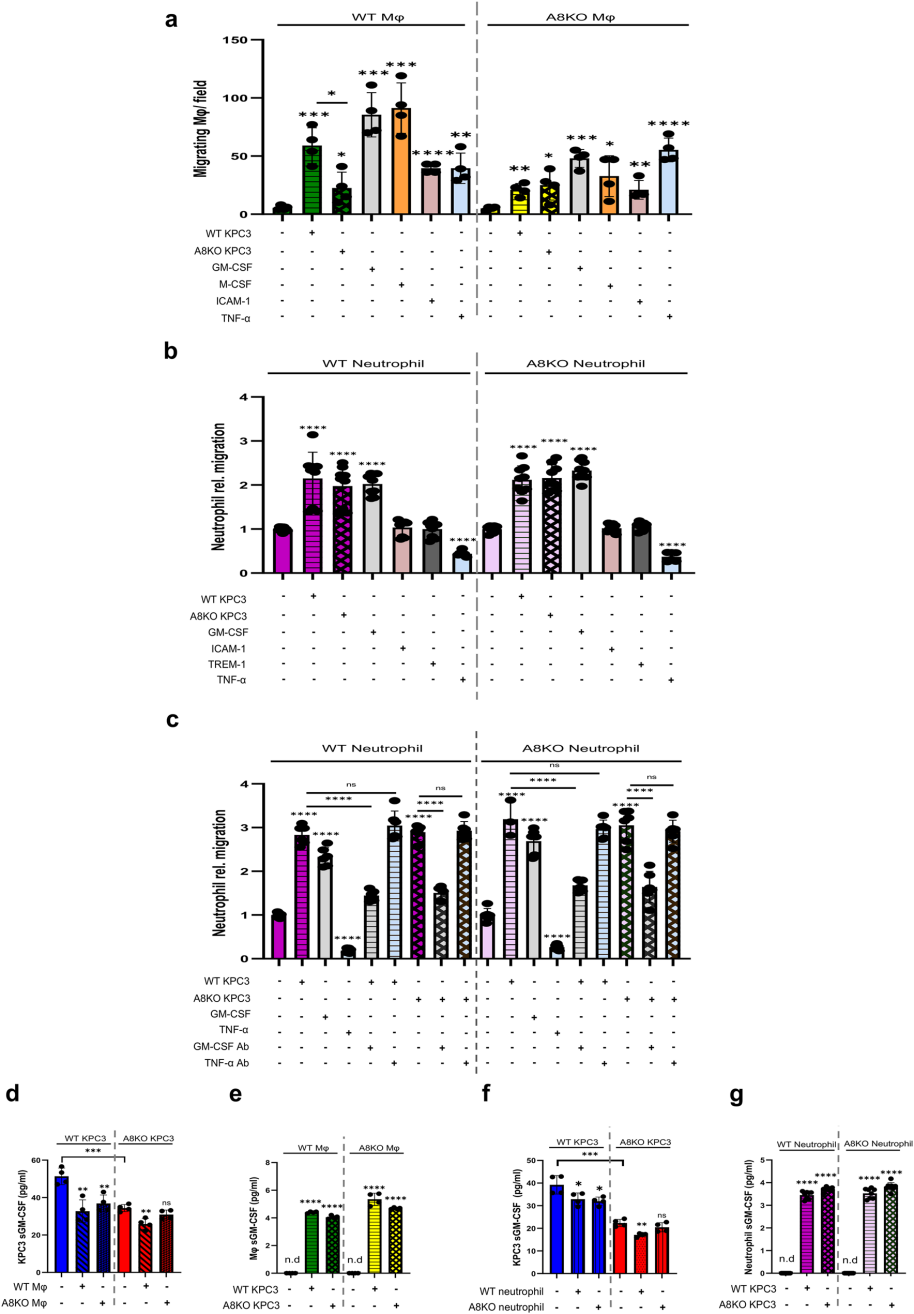
Role of ADAM8 in PDAC cell-mediated recruitment of macrophages and neutrophils. (**a**) Represents relative migration of WT/*A8*KO macrophages treated with WT/*A8*KO KPC3 cells, 50 ng/ml murine recombinant GM-CSF, M-CSF, ICAM-1 and TNF-α proteins compared to the control (untreated macrophages) (*n* = 2). Relative migration of WT/*A8*KO neutrophils after treatment with (**b**) The conditioned medium from WT/A8KO KPC3 (*n* = 3), 50 ng/ml murine recombinant GM-CSF (*n* = 3), ICAM-1 (*n* = 3), TREM-1 (*n* = 3) and TNF-α (*n* = 2) proteins; as well as (**c**) conditioned medium from WT KPC3, 50 ng/ml murine recombinant GM-CSF, TNF-α proteins (*n* = 2), combining WT KPC3 conditioned medium with 100 ng/ml neutralizing GM-CSF/TNF-α antibodies (*n* = 2), conditioned medium from *A8*KO KPC3 without and with combining with 100 ng/ml neutralizing GM-CSF/TNF-α antibodies (*n* = 2) are illustrated, respectively. ELISA results demonstrate the soluble GM-CSF level of (**d**) KPC3 cells after co-culture with macrophages (*n* = 2), (**e**) macrophages after co-culture with KPC3 cells (*n* = 2), (**f**) KPC3 cells after co-cultivation with neutrophils (*n* = 2) and (**g**) neutrophils after co-cultivation with PDAC cells (*n* = 2). The graphs in this figure are presented as mean values ± S.D, and as statistical methods, One-Way ANOVA and Student’s *t-test* were used to determine significance with * *p* < 0.05, ** *p* < 0.01, *** *p* < 0.001, **** *p* < 0.0001

To determine the functional impact of ADAM8 in PDAC cells, as well as ADAM8-regulated cytokines (according to proteome array results of KPC3 cells) on the migratory ability of macrophages in tumor microenvironment, we performed a transwell migration assay using WT/*A8*KO macrophages either with or without WT/*A8*KO KPC3 cells, 50 ng/ml recombinant GM-CSF, M-CSF, ICAM-1 and TNF-α proteins. The results revealed that, although the migratory capacity of macrophages significantly increased after treatments in both groups (WT and KO macrophages), the overall trend of this increase was lower across nearly all treatment conditions in *Adam8*-deficient macrophages compared to their wild-type counterparts. A closer examination of the data showed that while all treatments significantly enhanced the migration of macrophages, WT tumor cells, GM-CSF, and M-CSF elicited a more robust effect on the WT macrophage recruitment. However, GM-CSF and TNF-α proteins induced a greater increase in migration in *A8*KO macrophages compared to the other factors. Among the cytokines tested, TNF-α was the only one that stimulated migration more in KO macrophages than in WTs. This may be attributed to the higher TNF-α levels in the WT macrophage secretome, compared to the WTs, as indicated by the proteome array results, suggesting a possible dose-dependent effect of TNF-α. This finding underscores the essential role of ADAM8 in facilitating tumor- and cytokine-mediated macrophage recruitment (Fig. 6a).

Next, to investigate the influence of A8 in tumor cells as well as the impact of A8-regulated cytokines derived from PDAC cells and neutrophils on neutrophil migratory capability, we conducted a transwell migration assay using WT/*A8*KO neutrophils, under different treatment conditions including: WT/A8KO KPC3 cells, 50 ng/ml recombinant GM-CSF, ICAM-1, TREM-1, and TNF-α proteins. The results depicted that the migratory ability of the neutrophils was significantly increased in response to tumor cells and GM-CSF, independent of ADAM8 expression. Conversely, the cell motility was markedly decreased by TNF-α treatment in neutrophils (Fig. 6b). Regarding the strong effects of GM-CSF and TNF-α on neutrophil migratory capacity, we performed an additional transwell migration assay on neutrophils using tumor cells with or without adding 100 ng/ml neutralizing antibodies against GM-CSF or TNF-α, treatment with 50 ng/ml recombinant GM-CSF and TNF-α proteins, to elucidate whether the observed effects of tumor cells on neutrophil migration are mediated by the mentioned cytokines. The results demonstrated that by blocking GM-CSF protein in the secretome of the tumor cells, the recruitment ability of the tumor cells was significantly reduced, whereas blocking TNF-α in the tumor secretome could not alter the ability of PDAC cells to recruit the neutrophils. These data suggest that GM-CSF may plays a key role in mediating neutrophil migration through the PDAC cell secretome (Fig. 6c).

Subsequently, given that ADAM8-dependent cytokines appeared to influence macrophage and neutrophil migration, we examined their secretion levels more closely in both tumor cells and immune cells following co-culture. Due to low cytokine concentrations, most of these cytokines were undetectable with our available ELISA kits. However, we successfully quantified soluble GM-CSF (sGM-CSF), a cytokine which has been shown by some studies to be implicated in immune cell migration [23, 24]. The analysis of sGM-CSF in the conditioned media from co-cultures of KPC3 cells with macrophages exhibited a significant downregulation of sGM-CSF levels not only in *Adam8*-deficient KPC3 cells but also after co-culture across almost all the conditions (Fig. 6d). In contrast, in macrophages, a significant increase in Gm-CSF level following co-culture with tumor cells was observed (Fig. 6e). Additionally, sGM-CSF level from the co-culture of tumor cells with neutrophils showed almost the same pattern as that of the tumor cells and macrophages, meaning that it was considerably reduced in KO KPC3 cells as well as nearly all the co-culture conditions (Fig. 6f). However, neutrophils themselves exhibited a significant upregulation of sGM-CSF secretion after co-culture with PDAC cells, regardless of *Adam8* expression (Fig. 6g).

### Analysis of PDAC pathology and immune cell composition in pancreatic tissues of KPC mice with WT and *A8KO* background

**Fig. 7:**
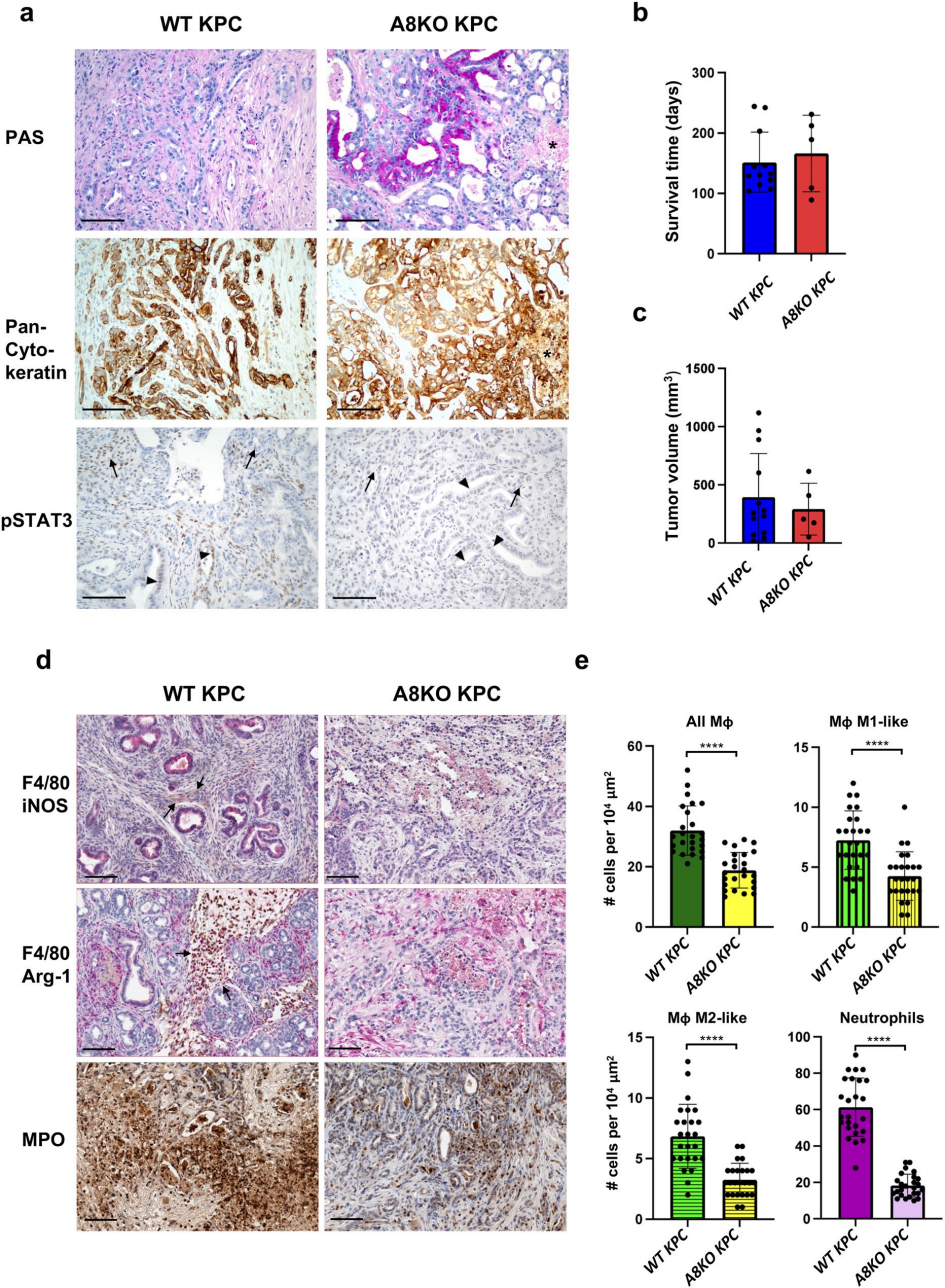
Evaluation of PDAC pathology and immune cell populations in pancreatic tissues of WT/A8KO KPC mice. (**a**) histology of WT KPC and A8KO KPC mice showing PDAC by PAS (Periodic acid-Schiff) stain, immunohistochemistry for Pan-cytokeratin (CKMNF116) with ductal adenocarcinoma and a strong desmoplastic stromal reaction and staining for phospho-STAT3. Note that the A8KO KPC mouse exerts ductal adenocarcinoma with partial mucinous differentiation as revealed in the PAS stain. Asterisks mark tumor necrosis, Arrowheads tumor cells, and arrows stroma cells. (**b**) Comparison of survival times of WT KPC and A8KO KPC mice. (**c**) Tumor sizes between the two groups of mice. (**d**) The first and second rows compare M1 and M2 like macrophages using Anti-F4/80 antibody as a general macrophage marker (brown signals), along with either anti-NOS or anti-Arg-1 antibodies (Magenta signals) to distinguish M1 and M2 subtypes, respectively, in the presence and absence of ADAM8. The third row shows staining of mouse pancreatic tissues with an anti-MPO antibody (brown signals) as a neutrophil marker. (**e**) Contains the quantification of the stainings, comparing total macrophage numbers, M1/M2-like macrophage population and neutrophil infiltration between the two groups. The graphs in this figure are presented as mean values ± S.D., and Student’s *t*-test was used for significance with * *p* < 0.05, ** *p* < 0.01, *** *p* < 0.001, **** *p* < 0.0001. Scale bar, all images, 100 μm

To investigate the effects of ADAM8 loss on mouse characteristics, WT KPC (*LSL-Kras*^*G12D/+*^*Trp53*^*R172H/+*^*Pdx-1-Cre)* mice were crossed with *A8KO* mice, generating some offspring with the genotype of *LSL-Kras*^*G12D/+*^*Trp53*^*R172H/+*^*Pdx-1-Cre Adam8*^-/+^ in the first generation. Next, these heterozygous mice were intercrossed, which resulted in homozygous *A8* knockout KPC mice in the F2 generation (Supplemental Figure S6a). Using specific primers targeting *Kras, Trp53, Cre and Adam8* genes and ear biopsies of the mice, genotyping was performed to identify the genotype of the mice for the next experiments (Figure S6b). A table summarizes breeding outcomes of WT and A8KO KPC mice, listing genotypes, survival, tumor burden, pancreatic weights, and clinical observations. A total of 32 mice were obtained (25 WT KPC and 7 A8KO KPC) (Table 1). Immunohistochemistry of pancreatic tissues confirmed the absence of ADAM8 in *A8*KO KPC mice, with clear differences from WT despite some background staining (Figure S6c). Immunostaining of representative sections from WT and A8KO KPC mice revealed a slightly higher proportion of mucinous differentiation (PAS staining) and enhanced pan-cytokeratin staining in A8KO KPC mice compared to WT KPC (Fig. 7a). Notably, staining for phospho-STAT3 was seen in tumor cells and in stromal immune cells, but with a lower frequency and intensity in A8KO KPC mice compared to the WT KPC mice (Fig. 7a). Quantitative analysis of survival data, as well as the pancreatic tumor size, indicated that the survival time of the KPC mice was not significantly affected by the presence of ADAM8 (Fig. 7b). Similarly, although some variability in tumor volume was observed, no notable differences were detected between the WT and *A8KO* groups (Fig. 7c), which is due to the heterogeneity of the KPC phenotype in WT KPC mice and to the low number of A8KO KPC mice that could be generated (Table 1). From all mice generated, five corresponding mice (see Table 1) were analyzed for tumor volumes and survival times with a significant trend to show reduced tumor volumes and increased survival times in KPC A8KO mice (Fig S7), however this finding is not sufficiently powered due to the low number of KPC A8KO mice generated over the breeding period and due to the heterogeneity of the observed tumor characteristics.

To investigate the major role of ADAM8 found in the co-culture experiments in modulating immune cell infiltration within the tumor microenvironment of PDAC in vivo, pancreatic tissues from WT and *A8KO* KPC mice were analyzed by immunohistochemistry. Tissue sections were co-stained with anti-F4/80 and either anti-iNOS or anti-Arg1 antibodies to identify M1-like and M2-like macrophages, respectively. In addition, neutrophils were detected using anti-MPO antibody staining. WT KPC tumors exhibited dense infiltration of macrophages with clear iNOS and Arg1 expression, while *A8KO* KPC tissues displayed notably reduced signals in all markers, indicating diminished macrophage presence and polarization (Fig. 7d). Quantitative analysis confirmed a significant reduction in the number of total macrophages, as well as M1- and M2-like subpopulations, in *A8KO* KPC tumors compared to WT controls (Fig. 7e). Furthermore, neutrophil infiltration, as indicated by MPO marker (Fig. 7d), was also significantly lower in *A8KO* KPC tissues (Fig. 7e). The number of T cells based on CD3 staining, were not significantly affected (Fig S8). These results suggest that ADAM8 promotes the recruitment and/or retention of both macrophages and neutrophils in the PDAC tumor microenvironment, thereby affecting the overall immune landscape of the tumor.

**Table 1:**
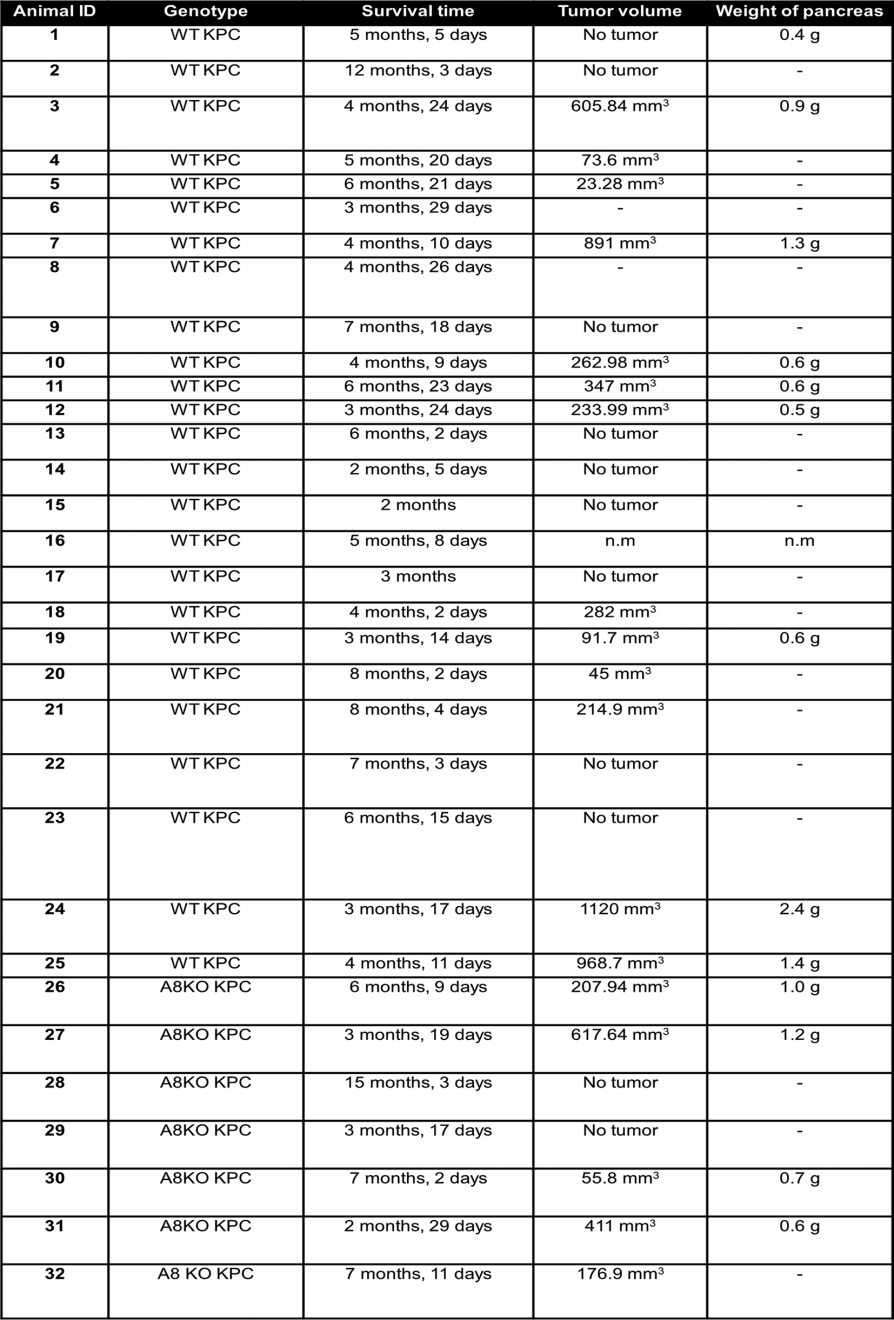
Results from breeding either A8WT (A8^+/+^)/KPC or A8KO (A8^-/-^)/KPC mice with survival times, tumor weight of the pancreas and the condition of these mice, based on weekly scoring and examinations. Note that mouse numbers 3 and 26; 7 and 31; 12 and 30; 25 and 32; 19 and 1 were littermates

### Discussion

The potential of ADAM8 as a therapeutic target in PDAC was recently highlighted by its elevated expression in tumor cells [12], and further investigations in PDAC tissue allowed to detect additional ADAM8 expression in cells of the TME [16]. Although previous studies have identified ADAM8 expression in the PDAC TME and provided limited evidence of its role, detailed information on the mechanisms of ADAM8 action in this microenvironment is still lacking. In the present study, we provide evidence that ADAM8 may significantly influence tumor characteristics and behavior, including immune cell recruitment, activation of pro-tumorigenic signaling cascades, and modulation of immune cell functions, thereby potentially contributing to a pro-tumoral PDAC TME. Our study integrated both in vitro and in vivo approaches, allowing us to examine the tumor-intrinsic and microenvironmental roles of ADAM8.

The results showed that in PDAC cells, high ADAM8 expression correlates with enhanced cell migration and proliferation. Similarly, in a study on human PDAC cell lines carrying a knockout of ADAM8, a substantial reduction in cell migration and invasion through extracellular matrix substrates was observed [25]. WT and A8KO bone marrow-derived macrophages and neutrophils showed no significant difference in migratory capacity under normal, unstimulated conditions. The proteome array results of our study revealed that ADAM8 is an important regulator of the secretion network in PDAC. In KPC3 cells ± ADAM8, secretion level of Granulocyte-Macrophage Colony-Stimulating Factor (GM-CSF) Macrophage Colony-Stimulating Factor (M-CSF), Intercellular Adhesion Molecule-1 (ICAM-1), and TNF-α (Tumor Necrosis Factor- alpha) were affected by ADAM8. Interestingly, this decrease was mirrored by reduced mRNA expression of Gm-csf, M-csf, Icam-1, and Tnf-α. This is an important finding, since cellular communication within the tumor microenvironment (TME) is predominantly governed by cytokines, chemokines, and other soluble factors. In PDAC, tumor-derived soluble factors can recruit and educate stromal cells, while signals from stromal cells in turn promote tumor progression [26]. A number of studies collectively show that the tumor microenvironment (TME), especially myeloid and stromal components, actively sustains pancreatic tumor growth by enforcing immunosuppression and shaping tumor cell programs.

For instance, GMCSF acts as a central tumorderived factor that programs inflammatory myeloid cells and thereby creates a chronic, tumorpromoting inflammatory milieu [27]. Across these studies, PDAC is portrayed as a TME-driven disease in which tumor derived factors (GMCSF, Cxcl1 [28]) recruit and program myeloid cells, while macrophages, neutrophils, and CAFs in turn enforce Tcell exclusion, sustain oncogenic transcriptional states, and limit treatment efficacy.

So far, no direct evidence has linked ADAM8 to GM-CSF, but ADAM8-regulated STAT3 activation may mediate this connection. Consistently, the proteome array of KPC3 cells lacking ADAM8 revealed downregulation of both IL-6 and GM-CSF, and western blot results showed the correlation between ADAM8 and STAT3. The mechanism by which ADAM8 regulates M-CSF is unclear, but it may act indirectly through ADAM17, the main sheddase of M-CSF, whose activity can be enhanced by ADAM8-mediated cleavage [28]. This cascade could explain the observed correlation between ADAM8 and M-CSF in our results. ADAM8 may also regulate ICAM-1 either by inducing transcription through STAT3 signaling via a miR-181a-5p/NEAT1 axis that stabilizes STAT3 and enhances ICAM-1 expression, promoting tumor progression [13], or by contributing to ICAM-1 shedding alongside ADAM17 [28]. However, similar to GM-CSF and M-CSF, ADAM8-dependent ICAM-1 reduction is more likely mediated at the mRNA level. Moreover, consistent with our findings on the correlation between ADAM8 and TNF-α, a study demonstrated that ADAM8 activity promotes the release of pro-inflammatory cytokines; silencing ADAM8 in microglial immune cells significantly diminishes TNF-α production (as well as IL-1β) upon inflammatory stimulation [29].

Furthermore, we examined the influence of ADAM8 on STAT3, FAK, and Src signaling, pathways recognized as key drivers of tumor progression, migration, and invasion in various cancer types [19–21], in both KPC3 and immune cells. The findings showed that the absence of ADAM8 significantly reduced phosphorylation of STAT3, FAK, and Src in tumor cells, as well as STAT3 in both macrophages and neutrophils. Part of these data aligns with previous studies examining the role of ADAM8 in regulating these proteins. For instance, as mentioned earlier, a recent study revealed that, in pancreatic cancer, ADAM8 modulates STAT3 signaling through the miR-181a-5p/NEAT1 axis. High ADAM8 expression leads to reduced miR-181a-5p levels, which increases NEAT1 expression, stabilizing STAT3 by preventing its degradation. This results in aberrant STAT3 activation, contributing to tumor progression [13]. In line with our findings, another report in pancreatic cancer indicated that ADAM8 can regulate FAK phosphorylation through its interaction with integrin β1, a receptor involved in cell adhesion and signaling. ADAM8 activation leads to increased FAK phosphorylation, which, in turn, promotes downstream signaling pathways that support cancer cell survival, migration, and invasion [12].

To investigate how ADAM8 influences cellular crosstalk and activates tumorigenic signaling pathways, we established 2D co-culture models. Of note, co-culture of ADAM8-expressing tumor cells with wild-type (WT) macrophages led to increased activation of STAT3, and to a lesser extent p-FAK, in PDAC cells. This suggests that signals from WT macrophages require ADAM8 in tumor cells to exert their effect, as no such response was observed in A8KO KPC3 cells co-cultured with WT macrophages. Moreover, our analysis revealed that in WT macrophages co-cultured with tumor cells, both ADAM8 mRNA and protein levels significantly increased. Additionally, the reduced STAT3 activation in KO macrophages was compensated when co-cultured with WT KPC3 cells.

Given the observed effects of ADAM8 on tumor–immune cell crosstalk through intercellular signaling activation, as well as its effect on cytokine secretion, we next investigated how these events influence immune cell recruitment in the PDAC TME. Scratch assays showed that the migration of both WT and KO macrophages increased when treated with WT or *A8*KO KPC3 cells, or with ADAM8-dependent cytokines (GM-CSF, M-CSF, ICAM-1, TNF-α). However, migration was consistently higher in WT macrophages, with WT KPC3 cells, GM-CSF, and M-CSF exerting the strongest effects. The lower trend of motility of KO macrophages under different conditions suggests that ADAM8 is critical for macrophage migration within the TME, likely due to reduced secretion of these cytokines by KO tumor cells. These results are in line with previous studies on the role of these cytokines in immune cell migration. For instance, GM-CSF plays a critical role in immune cell migration and tumor progression. In the context of monocytes, GM-CSF has been shown to enhance their migration across the blood-brain barrier, a process crucial in diseases like multiple sclerosis. This effect is mediated by the upregulation of adhesion molecules, such as LFA-1 and VLA-4, on monocytes, which facilitates their transmigration across endothelial cells [23]. As for M-CSF, it interacts with the CSF1 receptor (CSF1R) on monocytes and macrophages, promoting their differentiation into M2/TAM phenotypes, and triggers pathways through which tumors recruit tumor-associated macrophages (TAMs) and drive their polarization toward pro-tumoral functions [30]. A study by Liou et al. demonstrated that in pancreatic acinar cells, genetic alteration in KRAS can stimulate the expression of ICAM-1. This cytokine, in turn, acts as a chemoattractant for macrophages. The recruitment of macrophages leads to the secretion of TNF, which further promotes the transformation of acinar cells into duct-like structures (ADM) [31]. Regarding the role of TNF-α in macrophage migration, Vera et al. demonstrated that TNF-α stimulates the release of MCP1/CCL2, promoting macrophage migration in breast cancer. This process, driven by mitochondrial reactive oxygen species (mtROS), creates an inflammatory environment that may support tumor progression [32]. In terms of neutrophil migration, conditioned media from PDAC cells significantly enhanced migration compared to control conditions. Interestingly, this neutrophil-attracting effect of PDAC did not require tumour-based ADAM8, suggesting that PDAC cells secrete soluble factors to attract neutrophils largely independent of ADAM8 in vitro. Furthermore, the results showed that GM-CSF significantly enhanced neutrophil migration, whereas TNF-α reduced it. The effect of GM-CSF is consistent with previous studies, which have shown that GM-CSF serves as a potent chemoattractant for neutrophils, promoting their migration to sites of inflammation or infection. Tumor cells secrete GM-CSF, along with other factors such as IL-1β and CXCL chemokines, to recruit neutrophils into the tumor microenvironment. This recruitment can influence tumor progression, as neutrophils may either suppress or promote tumor growth depending on their polarization state and the cytokine environment [33]. In contrast, TNF-α treatment resulted in a reduction of neutrophil migration through the transwell. Although TNF-α can act as a chemoattractant in certain contexts, it more often amplifies inflammation rather than providing a direct gradient for chemotaxis. Lokuta and Huttenlocher demonstrated that TNF-α inhibits neutrophil migration and induces firm adhesion through the activation of the p38 MAPK pathway. This signaling cascade promotes the formation of vinculin-containing focal complexes, stabilizing neutrophil adhesion to surfaces like fibrinogen-coated substrates [34].

To validate our in vitro findings and assess the role of ADAM8 in PDAC progression in vivo, WT and A8KO KPC mice were generated and monitored for survival, tumor growth, pancreatic weights, and metastatic features. Overall, survival and tumor volume did not differ significantly between the two groups averaged over all mice obtained, but when individual littermates were compared, the trend to lower tumor load and longer survival of A8KO KPC mice was observed and is in agreement with earlier findings when beneficial effects of pharmacological inhibition of ADAM8 in KPC mice were reported (Fig S5; 12). Immunohistochemical analysis revealed significant effects on immune infiltration in the PDAC TME of A8KO KPC mice as both, the overall number of tumor-associated macrophages (TAMs), and their polarization toward M1 and M2 states were significantly reduced compared to WT tumors, suggesting that ADAM8 drives macrophage recruitment and functional polarization, likely through ADAM8-dependent cytokines. To the best of our knowledge, no other studies have specifically examined the role of ADAM8 in macrophage polarization. However, the relationship between M1/M2 macrophages and ADAM8 expression in pancreatic cancer has been studied. It was observed that M2 macrophages can induce ADAM8 expression in tumor cells, thereby increasing secretion of pro-tumorigenic factors such as LCN2 and MMP-9 [25]. Neutrophil infiltration (MPO^+^ cells) was also markedly reduced in A8KO tumors. This finding aligns with studies in inflammatory models, where ADAM8 inhibition reduced neutrophil penetration and tissue injury [35]. However, our in vivo results contradict our in vitro findings, which showed that ADAM8 in tumor cells is not required for neutrophil migration. This discrepancy may be due to the simplified in vitro setup, which relied on conditioned media and lacked direct tumor–immune cell interactions. Factors such as ICAM-1, which requires receptor-mediated binding (e.g., to LFA-1) to promote neutrophil infiltration, may not function properly under these conditions. Moreover, the complexity of the tumor microenvironment, with multiple interacting stromal and connective tissue components, cannot be fully captured in vitro. Finally, phospho-STAT3 staining appeared to be more numerous in WT KPC tumors, suggesting that the ADAM8-dependent STAT3 activation observed in co-culture experiments is reproducible in vivo and recapitulates the ADAM8 effect observed in vitro.

**Fig. 8:**
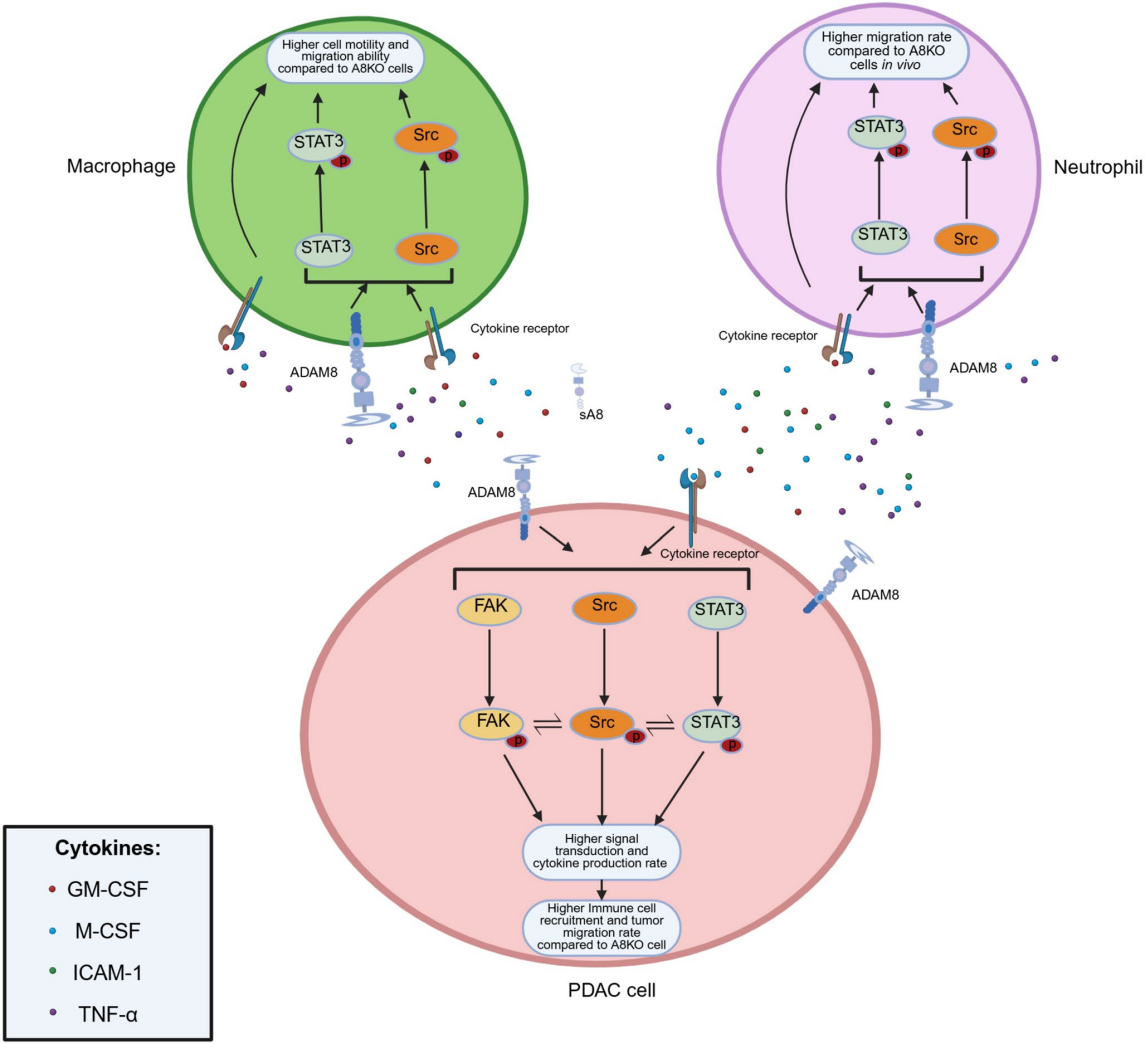
Graphical sketch of ADAM8 function in the PDAC microenvironment through interaction with immune cells. Briefly, in WT PDAC cells, ADAM8 regulates the secretion of pro-migratory cytokines, including GM-CSF, ICAM-1, M-CSF and TNF-α to the tumor microenvironment, as well as the activation of FAK/Src/STAT3 signalling pathways. This can promote enhanced tumor cell migration and proliferation compared to A8KO PDAC cells. Additionally, ADAM8 facilitates macrophage recruitment via regulating STAT3 and Src signalling, and also ADAM8 expression levels. Neutrophil migration is mainly driven by PDAC cells´ secretome, and cytokines such as GM-CSF. This process is regulated independently of ADAM8 in vitro, but in an ADAM8-dependent manner in vivo. On the other hand, macrophages and neutrophils can influence FAK/Src/STAT3 signalling in PDAC cells through the secretion of various cytokines or soluble factors into the microenvironment, altering the signal transduction patterns within the PDAC cells

In conclusion, our findings are summarized (Fig. 8) and suggest that ADAM8 may play an important role in both tumor-intrinsic behavior and the modulation of the PDAC microenvironment. Loss of ADAM8 impaired tumor cell migration and proliferation, likely through reduced activation of Src, FAK, and STAT3 signaling, pointing to a potential upstream role in pathways governing motility and invasion. In addition, ADAM8 deficiency was associated with lower expression of cytokines such as GM-CSF, M-CSF, ICAM-1, and TNF-α, which could affect immune cell recruitment and function. In vivo, ADAM8-deficient tumors showed reduced macrophage infiltration and polarization toward both M1 and M2 phenotypes, along with fewer neutrophils, supporting a potential role for ADAM8 in shaping immune cell dynamics within the TME.

## Electronic supplementary material

Below is the link to the electronic supplementary material.

Supplementary Material 1

## Data Availability

The datasets generated during and/or analyzed during the current study are available from the corresponding author on reasonable request.

## Abbreviations

PDAC: Pancreatic Ductal Adenocarcinoma
TME: Tumor Microenvironment
TAM: Tumor-Associated Macrophage
TAN: Tumor-Associated Neutrophil
ADAM8: A Disintegrin and Metalloproteinase 8
WT: Wild-Type
A8KO: ADAM8 Knockout
KPC: Kras^G12D/+^; Trp53^R172H/+^; Pdx-1-Cre mouse model
FAK: Focal Adhesion Kinase
Src: Proto-Oncogene Tyrosine-Protein Kinase Src
STAT3: Signal Transducer and Activator of Transcription 3
GM-CSF: Granulocyte–Macrophage Colony-Stimulating Factor
M-CSF: Macrophage Colony-Stimulating Factor
ICAM-1: Intercellular Adhesion Molecule 1
TNF-α: Tumor Necrosis Factor Alpha
TGF-β: Transforming Growth Factor Beta
ERK: Extracellular Signal-Regulated Kinase
qPCR: Quantitative Polymerase Chain Reaction
ELISA: Enzyme-Linked Immunosorbent Assay
PBS: Phosphate-Buffered Saline
FBS: Fetal Bovine Serum
DMEM: Dulbecco’s Modified Eagle Medium
HRP: Horseradish Peroxidase
SDS-PAGE: Sodium Dodecyl Sulfate–Polyacrylamide Gel Electrophoresis
FFPE: Formalin-Fixed, Paraffin-Embedded
DAB: 3,3′-Diaminobenzidine
PFA: Paraformaldehyde
RIPA: Radioimmunoprecipitation Assay Buffer
TBST: Tris-Buffered Saline with Tween-20
NP-40: Nonidet P-40 (nonionic detergent)
EDTA: Ethylenediaminetetraacetic Acid
BCA: Bicinchoninic Acid
CO₂: Carbon Dioxide
SD: Standard Deviation
H&E: Hematoxylin and Eosin
Q-VD-OPH: Quinoline-Val-Asp(Ome)-CH₂-O-Ph (a caspase inhibitor)
3-MA: 3-Methyladenine
DMSO: Dimethyl Sulfoxide
PCR: Polymerase Chain Reaction
RNA: Ribonucleic Acid
DNA: Deoxyribonucleic Acid
MAPK: Mitogen-Activated Protein Kinase
Mφ: Macrophage
KO: Knockout
HB-EGF: Heparin-Binding Epidermal Growth Factor
EGFR: Epidermal Growth Factor Receptor
CCL2: Chemokine (C-C motif) Ligand 2
VEGF: Vascular Endothelial Growth Factor
MMP-9: Matrix Metalloproteinase 9
ROS: Reactive Oxygen Species
DEPC: Diethyl Pyrocarbonate
PAGE: Polyacrylamide Gel Electrophoresis
SEM: Standard Error of the Mean
