# Supplementary material for: Deciphering the regulatory role of ADAM8 in the PDAC tumor microenvironment
Source: Cell Oncol (Dordr). 2026 Aug 3;49(4):101. doi: 10.1007/s13402-026-01266-7 (PMC13433674; doi:10.1007/s13402-026-01266-7)
Supplement: Supplementary file 1 [file 13402_2026_1266_MOESM1_ESM.docx]

**Supplementary figures, Zandieh et al.**

**a**

**b**

**b**

**Fig S1)** (a) **Mouse Cytokine Array** Panel A template containing 40 different proteins, 4 reference spots and 4 negative controls. (b) The table includes the information about the coordination of the targets/controls and their alternative nomenclature.

**Fig S2)** Proliferation assay results of WT/A8KO KPC3 cells after 24 hours under the same conditions as the wound healing assay for these cells. This experiment serves as a control for the scratch assay (*n* = 2).

**Fig. S3) Effects of apoptosis and autophagy inhibition on the proliferation rate of murine WT/A8KO PDAC cells. (a, b)** Depict the proliferation rate of WT/A8KO KPC3 cells following inhibition of apoptosis using Q-VD-OPH drug after 24 h and 72 h, respectively. **(c, d)** Show growth rate of WT/A8KO PDAC cells subsequent to inhibition of autophagy by 3-MA drug after 24 h and 72 h, respectively (n = 1). Data are presented as mean values ± S.D.

**Fig. S4) Evaluation of the invasiveness of murine WT/*A8*KO PDAC cells.** **(a, b)** Illustrates invasion assay results of WT/*A8*KO KPC3 cells after 24 h (n = 2).

**b**

**a**

**A8KO KPC3+A8KO Mφ**

**A8KO KPC3+WT Mφ**

**WT KPC3+A8KO Mφ**

**WT KPC3+WT Mφ**

**WT KPC3**

**A8KO KPC3**

**24 hours**

**0 hour**

**Fig S5) Analysis of KPC3 cell migration** in the presence of either WT of Adam8 KO macrophages (n=2).

**Fig. S6) Characterizing A8KO KPC mice as an *in vivo* model for PDAC. (a)** Exhibits an example of genotyping mouse biopsies from second-generation offspring to detect *Kras, Trp53, Cre* and *Adam8* alleles. **(b)** Immunohistochemistry results from the pancreatic tissue of WT/*A8*KO KPC mice to analyse ADAM8 protein using Hematoxylin (blue) and anti-ADAM8 Antibody; scale bar 250 µm.

**A	B	C	D**

**Fig S7) Analyses of tumor volumes and survival in WT KPC and A8KO KPC mice.** Comparison of 5 corresponding littermates KPC WT (blue) and KPC A8KO (red) with regard to tumor volumes (A,B) and survival time (C,D).

**Fig S8) T-cell infiltration of WT KPC vs. A8KO KPC mice.** (A) Immunohistochemistry for CD3 of PDAC from KPC WT mouse showing peripherally accented infiltrates of T lymphocytes. (B) Immunohistochemistry for CD3 of PDAC from KPC mouse with ADAM 8 KO showing slightly reduced peripherally accented infiltrates of T lymphocytes. Scale bar 100 µm.
